# HPLC-DAD-Guided Isolation of Diversified Chaetoglobosins from the Coral-Associated Fungus *Chaetomium globosum* C2F17

**DOI:** 10.3390/molecules25051237

**Published:** 2020-03-09

**Authors:** Xiao-Wei Luo, Cheng-Hai Gao, Hu-Mu Lu, Jia-Min Wang, Zi-Qi Su, Hua-Ming Tao, Xue-Feng Zhou, Bin Yang, Yong-Hong Liu

**Affiliations:** 1 Institute of Marine Drugs, Guangxi University of Chinese Medicine, Nanning 530200, China; luoxiaowei1991@126.com (X.-W.L.); gaochh@gxtcmu.edu.cn (C.-H.G.); lhm098@126.com (H.-M.L.); 2 CAS Key Laboratory of Tropical Marine Bio-resources and Ecology/Guangdong Key Laboratory of Marine Materia Medica, South China Sea Institute of Oceanology, Chinese Academy of Sciences, Guangzhou 510301, China; wjm2275322055@163.com (J.-M.W.); xfzhou@scsio.ac.cn (X.-F.Z.); 3 School of Traditional Chinese Medicine, Southern Medical University, Guangzhou 510515 China; ziqisl@163.com (Z.-Q.S.); taohm@smu.edu.cn (H.-M.T.)

**Keywords:** coral, marine fungi, *Chaetomium globosum*, cytochalasans, chaetoglobosins, cytotoxicity

## Abstract

Cytochalasans have continuously aroused considerable attention among the chemistry and pharmacology communities due to their structural complexities and pharmacological significances. Sixteen structurally diverse chaetoglobosins, 10-(indol-3-yl)-[13]cytochalasans, including a new one, 6-*O*-methyl-chaetoglobosin Q (**1**), were isolated from the coral-associated fungus *Chaetomium globosum* C2F17. Their structures were accomplished by extensive spectroscopic analysis combined with single-crystal X-ray crystallography and ECD calculations. Meanwhile, the structures and absolute configurations of the previously reported compounds **6**, **12**, and **13** were confirmed by single-crystal X-ray analysis for the first time. Chaetoglobosins E (**6**) and Fex (**11**) showed significant cytotoxicity against a panel of cancer cell lines, K562, A549, Huh7, H1975, MCF-7, U937, BGC823, HL60, Hela, and MOLT-4, with the IC_50_ values ranging from 1.4 μM to 9.2 μM.

## 1. Introduction

Corals generally constitute a dominant part of the reef biomass in tropical marine ecosystems, which are known to harbor diverse and highly abundant microbial communities such as fungi, bacteria, actinomycetes, and cyanobacteria [1]. In recent decades, coral-associated microorganisms have been considered to be extraordinary sources of bioactive natural products, such as phenolics, quinones, and alkaloids. Meanwhile, as an important component of corals, 95 different fungal species of 44 genera from 38 different coral strains have been cultured, including the common fungal genera of *Aspergillus* and *Penicillium*, and rare genera like *Bipolaris*, *Candida*, *Chaetomium* [2]. Fungi of the *Chaetomium* belong to the large genera of *Chaetomiaceae* family with more than 350 species, which have been reported as a prolific source of diversiform natural products, such as azaphilones, terpenoids, steroids, chaetoglobosins, xanthones, etc. [3].

Cytochalasans are a class of fungal alkaloids composed of a highly substituted perhydro-isoindolone moiety incorporating a macrocyclic ring (either a carbocycle, a lactone or a cyclic carbonate) that are assembled by polyketide synthase-nonribosomal peptide synthetase (PKS-NRPS) [4]. Variations in types of amino acids and the substitution patterns of the macrocycle would further highly extend the chemical diversity. Recently, a variety of cycloaddition heterodimers of cytochalasans with novel complicated architectures have been reported, exemplified by asperflavipine A [5], aureochaeglobosins A−C [6]. Chaetoglobosins, 10-(indol-3-yl)-[13]cytochalasans were recently reported to have cytotoxicity [6,7] and antibacterial activity [8], and have attracted great interest from both chemists and pharmacologists [9,10,11,12].

As part of our program to discover structurally novel and biologically significant secondary metabolites from marine fungi [13,14], our attention was drawn to the fungus *Chaetomium globosum* C2F17 derived from the coral *Pocillopora damicornis*, collected in the South China Sea. The HPLC with a diode-array detector (HPLC-DAD) analysis (Figure S12) of its EtOAc extract based on solid rice fermentation combined with our in-house UV spectra library displayed the characteristic UV absorption bands at around 205, 220, and 275 nm, revealing the presence of a series of indole alkaloids.

## 2. Results and Discussion

**Figure 1:**
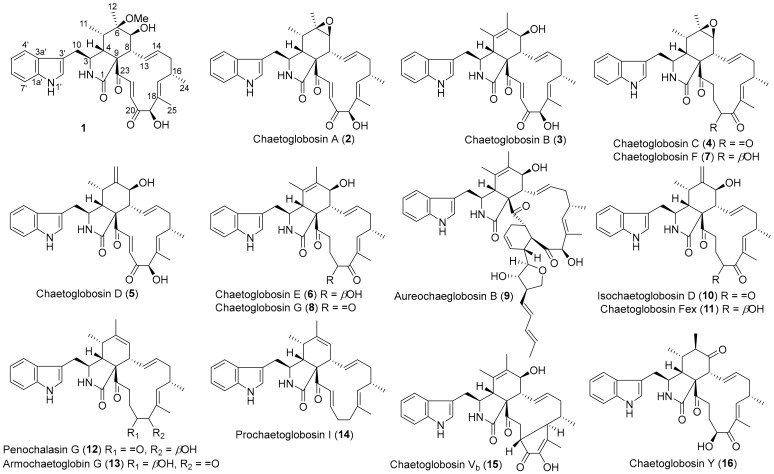
Chemical structures of compounds **1**‒**16**.

The rice fermentation broth of *C. globosum* C2F17 was extracted with EtOAc for three times. The whole extract was then partitioned and purified by repeated column chromatography involving silica gel, reversed-phase silica gel C18, and semipreparative HPLC. The HPLC-DAD-guided purification led to the discovery of 16 diversified chaetoglobosin derivatives (Figure 1).

### 2.1. Structural Elucidation

**Figure 2:**
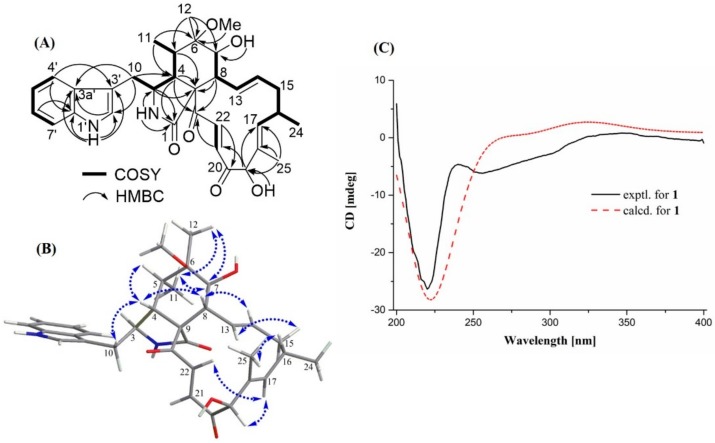
Key HMBC, COSY (**A**), and NOESY (**B**) correlations, and the experimental and calculated ECD spectra (**C**) of **1**.

Compound **1** was isolated as a white power with the molecular formula of C_33_H_40_N_2_O_6_ as determined by HRESIMS peak at *m/z* 561.2958 [M + H]^+^ (calcd for C_33_H_41_N_2_O_6_, 561.2965). The ^1^H-NMR data (Table 1) aided with HSQC spectrum of **1** revealed the typical characteristic of a chaetoglobosin scaffold with four exchangeable protons, ascribed to two NH groups [1′-NH (δ_H_ 10.86), 2-NH (δ_H_ 8.13)] and two hydroxyl groups [7-OH (δ_H_ 4.13, d, *J* = 8.4 Hz), 19-OH (δ_H_ 5.22, d, *J* = 4.9 Hz)], eight olefinic or aromatic protons, assigned to H-13 (δ_H_ 6.02, d, *J* = 14.0, 10.5 Hz), H-14 (δ_H_ 4.93, m), H-17 (δ_H_ 5.45, d, *J* = 9.1 Hz), H-2′ (δ_H_ 7.08, d, *J* = 2.1 Hz), H-4′ (δ_H_ 7.46, d, *J* = 8.4 Hz), H-5′ (δ_H_ 6.95, t, *J* = 7.7 Hz), H-6′ (δ_H_ 7.04, d, *J* = 7.7 Hz), and H-7′ (δ_H_ 7.31, d, *J* = 8.4 Hz), seven methines, H-3 (δ_H_ 3.44, m), H-4 (δ_H_ 2.91, dd, *J* = 7.0, 2.1 Hz), H-5 (δ_H_ 1.98, m), H-7 (δ_H_ 3.24, d, *J* = 4.9 Hz), H-8 (δ_H_ 2.27, t, *J* = 10.5 Hz), H-16 (δ_H_ 2.53, overlapped), and H-19 (δ_H_ 4.95, d, *J* = 4.9 Hz), two methylenes, H_2_-10 (δ_H_ 2.61, dd, *J* = 14.7, 6.3 Hz; 2.51, overlapped) and H_2_-15 (δ_H_ 2.24, m), one oxymethyl (δ_H_ 3.04, s), and four methyls, attributed to two singlets H_3_-12 (δ_H_ 1.13), H_3_-18 (δ_H_ 1.31), and two doublets H_3_-11 (δ_H_ 0.85, d, *J* = 6.3 Hz) and H_3_-16 (δ_H_ 0.95, t, *J* = 6.3 Hz). Apart from the aforementioned 24 corresponding carbons, nine nonprotonated ones remained in the ^13^C-NMR spectrum, including three carbonyls (δ_C_ 173.6, 199.3, 200.7), four olefinics (δ_C_ 109.9, 127.3, 132.5, 136.1), and two oxygenated quaternary ones (δ_C_ 61.9, 76.9). The above NMR data highly resembled those of chaetoglobosin Q excreted from *C. globosum* [15]. The only obvious difference was the occurrence of an additional oxygenated methyl (δ_H/C_ 3.04/49.2) located at C-6 in **1**, rather than a hydroxyl group in chaetoglobosin Q, which was also verified by the HMBC correlation (Figure 2) from H_3_-26 to C-6 (δ_C_ 76.9). The assignments of all carbon and proton resonances (Table 1) of **1** were finally made on the analysis of the HSQC, COSY, and HMBC experimental data.

**Figure 3:**
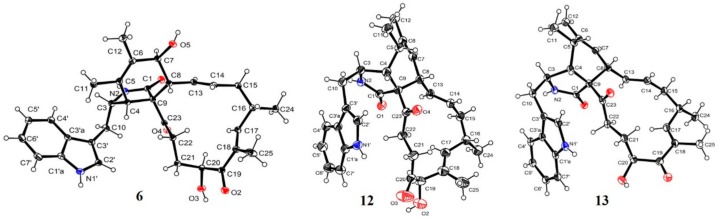
X-ray structures of chaetoglobosin E (**6**), penochalasin G (**12**), and armochaetoglobin G (**13**).

The relative configuration of **1** was deduced according to proton coupling constants (Table 1) and NOESY experiments (Figure 2), which led to the assignment of the sharing *E* configuration of the Δ^13^, Δ^17^, and Δ^21^ double bonds. The NOESY correlations of H-4/H-5, H-8, H_2_-10, and H-7/H_3_-11, H_3_-12 suggested that this set of protons (H-4, H-5, H-8, H_2_-10, and H_3_-26) was positioned on the same face. The same relative configuration and similar specific options between **1** ([*α*${]}_{D}^{25}$ −85 (*c* 0.05, MeOH)) and chaetoglobosin Q ([*α*${]}_{D}^{20}$ −100 (*c* 0.10, MeOH)) indicated they also shared an identical absolute configuration [15]. Moreover, the experimental ECD curve of **1** showed good agreement with the calculated ECD one (Figure 3), further confirming the above deduction. Taken together, the complete structure of **1** was established and accordingly assigned as 6-*O*-methyl-chaetoglobosin Q, which was probably obtained as an artifact from acidic solvolisis of the epoxide of chaetoglobosin A (**2**).

Additionally, the structures of these known compounds (**2**‒**16**) were mainly elucidated by spectral data analysis as well as comparison with those reported in the literature. They were identified as chaetoglobosins A‒G (**2**‒**8**) [16,17,18], aureochaeglobosin B (**9**) [6], isochaetoglobosin D (**10**) [18], chaetoglobosin Fex (**11**) [16], penochalasin G (**12**) [19], armochaetoglobin G (**13**) [7], prochaetoglobosin I (**14**) [8], chaetoglobosins V_b_ (**15**) [8] and Y (**16**) [18], respectively (Figure 1). Structurally, they shared a 3-substituted indole moiety and a 13-membered carbocyclic ring system with characteristic UV absorptions at around 205, 220, and 275 nm. Among them, aureochaeglobosin B was recently reported as a rare [4 + 2] cycloaddition heterodimer of chaetoglobosin and aureonitol. Of note, the X-ray single crystal structures and absolute configurations of chaetoglobosin E, penochalasin G, and armochaetoglobin G were firstly incontrovertibly confirmed by single-crystal X-ray crystallography herein (Figure 3).

### 2.2. Biological Activity

To our knowledge, there have been more than 300 cytochalasans from fungal sources reported with diverse structures and bioactivities [6]. The fungus *C. globosum* has been proven to be a rich source of cytochalasans. In addition, chaetoglobosins were recently found with cytotoxic [6,20,21], antibacterial [8], and phytotoxic activity [16], as well as immunomodulatory properties [22]. With this in mind, compounds **3**−**16** were screened for their cytotoxicity against a panel of cancer cell lines, H1975, U937, K562, BGC823, MOLT-4, MCF-7, A549, Hela, HL60, and Huh-7. Among them, chaetoglobosin E (**6**) showed significant cytotoxicity against K562, A549, Huh7, H1975, MCF-7, U937, BGC823, HL60, Hela, and MOLT-4 cell lines, with IC_50_ values of 8.9, 5.9, 1.4, 9.2, 2.1, 1.4, 8.2, 2.5, 2.8, and 1.4 μM, respectively. Additionally, chaetoglobosin Fex (**11**) showed selective cytotoxic activity against Huh7, MCF-7, U937, and MOLT-4 cell lines, with IC_50_ values of 3.0, 7.5, 4.9, and 2.9 μM, respectively. However, the remaining compounds were found to be inactive (IC_50_ > 10 μM) against the above cell lines. Furthermore, compounds **3**−**16** were also tested for the anti-tuberculosis activity, while none of them exhibited antituberculosis activity (MIC > 50 μM).

## 3. Materials and Methods 

### 3.1. General Experimental Procedures

Optical rotations were acquired by an Anton Paar MPC 500 polarimeter (Anton Paar, Graz, Austria). UV and IR spectra were recorded on a Shimadzu UV-2600 PC spectrometer and an IR Affinity-1 spectrometer, respectively (Shimadzu Corporation, Nakagyo-ku, Kyoto, Japan). ECD spectra were measured with a Chirascan circular dichroism spectrometer (Applied Photophysics Ltd., Leatherhead, UK). The NMR spectra were obtained on a Bruker Avance spectrometer (Bruker BioSpin, Fällanden, Switzerland) operating at 500 MHz or 700 MHz for ^1^H-NMR, 125 MHz or 175 MHz for ^13^C NMR, using TMS as an internal standard. HR-ESIMS spectra were collected on a Bruker miXis TOF-QII mass spectrometer (Bruker BioSpin, Fällanden, Switzerland). X-ray diffraction intensity data were performed on an XtaLAB PRO single-crystal diffractometer using Cu K*α* radiation (Rigaku, Japan). TLC and column chromatography (CC) were performed on plates precoated with silica gel GF254 (10–40 μM) and over silica gel (200–300 mesh) (Qingdao Marine Chemical Factory, China), respectively. All solvents employed were of analytical grade (Tianjin Damao Chemical and Industry Factory, Tianjin, China). Semi-preparative HPLC was performed on a Hitachi Primaide (Hitachi, Tokyo, Japan) using an ODS column (YMC-pack ODS-A, YMC Co. Ltd., Kyoto, Japan, 10 × 250 mm, 5 μM, 2.5 mL/min). The artificial sea salt was a commercial product (Guangzhou Haili Aquarium Technology Company, Guangzhou, China).

### 3.2. Fungal Strain and Fermentation

The strain C2F17 was isolated from a coral *Pocillopora damicornis*, which was collected from the seashore near Sanya Bay, Hainan Province, China, in March 2018. The isolates were stored on Müller Hinton broth (MB) agar (malt extract 15 g, artificial sea salt 15 g, and agar 20 g in 1.0 L tap distilled H_2_O) slants at 4 °C, and a voucher specimen was deposited in our lab. It was identified as *Chaetomium globosum* C2F17 based on sequence analysis of the internal spacer (ITS) regions of the rDNA (GenBank accession no. MN826318, Figure S13). The strain *C. globosum* C2F17 was cultured on MB-agar plates at 25 °C for 7 days. Then, it was inoculated in the seed medium (malt extract 15 g and artificial sea salt 15 g in 1.0 L tap distilled H_2_O, pH 7.4–7.8) at 25 °C for 48 h under shaking conditions (180 rpm). Subsequently, a large-scale fermentation of *C. globosum* C2F17 was performed in modified rice solid medium (120 g rice, 2.4 g artificial sea salt, 1.0 g bacteriological peptone, and 150 mL H_2_O) employing with 1 L × 49 Erlenmeyer flasks at room temperature under static conditions for 45 days. All of the fermented cultures were overlaid and extracted with EtOAc three times to afford a brown extract (120 g).

### 3.3. Extraction and Isolation

The EtOAc extract was firstly fractionated by silica gel vacuum liquid chromatography (VLC) using a step gradient elution with petroleum ether/CH_2_Cl_2_ (0~100%), which afforded ten fractions (Frs.1~10) based on TLC (GF_254_) properties. Fr.8 was further separated into twenty-five subfractions (Frs.8-1~8-25) via ODS silica gel chromatography eluting with MeCN/H_2_O (10~100%). Fr.8-9 was purified by semipreparative HPLC (48% MeCN/H_2_O, 2 mL/min, 220 nm) to afford **5** (5 mg, t_R_ 20 min), **3** (45 mg, t_R_ 22 min), **1** (2 mg, t_R_ 29 min), **14** (3.7 mg, t_R_ 32 min), and **9** (3.2 mg, t_R_ 35 min). Fr.8-16 was directly separated by semipreparative HPLC (55% MeCN/H_2_O, 2 mL/min, 220 nm) to yield **6** (13 mg, t_R_ 13 min), **15** (9 mg, t_R_ 15 min), **4** (15 mg, t_R_ 29 min), **13** (4 mg, t_R_ 44 min), **12** (10 mg, t_R_ 48 min), and two sub-fractons, Fr.8-16-4 (t_R_ 16 min) and Fr.8-16-5 (t_R_ 20 min), respectively. Additionally, Fr.8-16-4 was purified by semipreparative HPLC (75% MeOH/H_2_O, 2 mL/min, 220 nm) to offer **10** (10 mg, t_R_ 11 min), **7** (120 mg, t_R_ 13 min), and **11** (14 mg, t_R_ 15 min). Fr.8-16-5 was purified by semipreparative HPLC (50% MeOH/H_2_O, 2 mL/min, 220 nm) to provide **8** (4 mg, t_R_ 11 min), **2** (3 mg, t_R_ 14 min), and **16** (3 mg, t_R_ 16 min).

6-*O*-methyl-chaetoglobosin Q (**1**). white amorphous powder; [α${]}_{D}^{25}$ −85 (c 0.05, MeOH); UV (MeOH) λ_max_ (logε) 203 (4.15), 221 (4.24), 280 (3.44), 290 (3.37) nm; ECD (0.30 mg/mL, MeOH) λ_max_ (Δε) 220 (−15.02), 240 (−2.65), 247 (+0.47) nm; IR (film) ν_max_ 3334, 2949, 1633, 1681, 1456, 1203, 1141, 1020 cm^−1^; ^1^H and ^13^C NMR data, Table 1; HR-ESIMS m/z 561.2958 [M + H]^+^ (calcd for C_33_H_41_N_2_O_6_, 561.2965), 583.2775 [M + Na]^+^ (calcd for C_33_H_40_N_2_NaO_6_, 583.2784).

### 3.4. Computational Methods

The theoretical ECD curve of **1** was calculated by the Gaussian 09 software (Gaussian, Inc., Wallingford, CT, USA). Conformational searches were carried out by means of the Spartan’14 software (Wavefunction Inc., Irvine, CA, USA) using a Molecular Merck force field (MMFF) [14]. Low-energy conformers with a Boltzmann distribution over 1% were chosen for ECD calculations by TD*-*DFT method at the B3LYP/6*-*311+G (d, p)//B3LYP/6*-*31+G (d) level in methanol by adopting 30 excited states. The ECD spectra were generated by the SpecDis 3.0 (University of Würzburg, Würzburg, Germany) under a half bandwidth of 0.4 eV and shifted by 25 nm to facilitate comparison to the experimental data.

### 3.5. X-Ray Crystallography

The crystallographic data of compounds **6**, **12**, and **13** obtained in MeOH were collected on a Rigaku XtaLAB PRO single-crystal diffractometer using Cu K*α* radiation. Briefly, their X-ray crystal structures were solved using SHELXS97, expanded by difference Fourier techniques, and refined by full-matrix least-squares calculation finally. The non-hydrogen atoms were refined anisotropically, and hydrogen atoms were fixed at calculated positions.

Crystal data of chaetoglobosin E (**6**): C_32_H_38_N_2_O_5_, *M*r = 530.64, crystal size 0.2 × 0.1 × 0.05 mm^3^, monoclinic, *a* = 7.72270(10) Å, *b* = 14.66050(10) Å, *c* = 12.33200(10) Å, *α* = 90°, *β* = 99.2980(10)°, *γ* = 90°, *V* = 1377.87(2) Å^3^, T = 104(6) K, space group *P*2_1_, *Z* = 2, μ(CuK*α*) = 0.692 mm^−1^, 16,008 reflections collected, 5168 independent reflections (*R*_int_ = 0.0305). The final *R*_1_ values were 0.0375 (I > 2*σ*(I)). The final w*R*(F^2^) values were 0.1035 (I > 2*σ*(I)). The final *R*_1_ values were 0.0381 (all data). The final w*R*(F^2^) values were 0.1038 (all data). The goodness of fit on F^2^ was 1.074. The Flack parameter was −0.10(8). The crystallographic data for the structure of **6** have been deposited in the Cambridge Crystallographic Data Centre (deposition number: CCDC 1981873).

Crystal data of penochalasin G (**12**): C_32_H_38_N_2_O_4_·H_2_O, *M*r = 532.66, crystal size 0.1 × 0.08 × 0.06 mm^3^, monoclinic, *a* = 13.7791(10) Å, *b* = 7.6019(5) Å, *c* = 14.2066(12) Å, *α* = 90°, *β* = 105.358(8)°, *γ* = 90°, *V* = 1434.96(19) Å^3^, T = 105(8) K, space group *P*2_1_, *Z* = 2, μ(CuK*α*) = 0.665 mm^−1^, 15,184 reflections collected, 5454 independent reflections (*R*_int_ = 0.0402). The final *R*_1_ values were 0.0681 (I > 2*σ*(I)). The final w*R*(F^2^) values were 0.1859 (I > 2*σ*(I)). The final *R*_1_ values were 0.0839 (all data). The final w*R*(F^2^) values were 0.1994 (all data). The goodness of fit on F^2^ was 1.074. The Flack parameter was −0.01(15). The crystallographic data for the structure of **12** have been deposited in the Cambridge Crystallographic Data Centre (deposition number: CCDC 1971980).

Crystal data of armochaetoglobin G (**13**): C_34_H_46_N_2_O_6_·2CH_3_OH, *M*r = 578.73, crystal size 0.12 × 0.04 × 0.04 mm^3^, monoclinic, *a* = 11.7862(8) Å, *b* = 7.4775(5) Å, *c* = 18.2085(14) Å, *α* = 90°, *β* = 103.022(8)°, *γ* = 90°, *V* = 1563.5(2) Å^3^, T = 100(1) K, space group *P*2_1_, *Z* = 2, μ(CuK*α*) = 0.672 mm^−1^, 15,447 reflections collected, 6057 independent reflections (*R*_int_ = 0.0505). The final *R*_1_ values were 0.0581 (I > 2*σ*(I)). The final w*R*(F^2^) values were 0.1595 (I > 2*σ*(I)). The final *R*_1_ values were 0.0643 (all data). The final w*R*(F^2^) values were 0.1627 (all data). The goodness of fit on F^2^ was 1.116. The Flack parameter was 0.3(2). The crystallographic data for the structure of **13** have been deposited in the Cambridge Crystallographic Data Centre (deposition number: CCDC 1981877).

### 3.6. Cytotoxicity Assay

Compounds **3**−**16** were tested for their cytotoxicity against several cancer cell lines, H1975 (human lung adenocarcinoma), U937 (human lymphocytic leukemia), K562 (human chronic myelogenous leukemia), BGC823 (human gastric adenocarcinoma), MOLT-4 (human acute T lymphoblastic leukemia), MCF-7 (human breast carcinoma), A549 (human lung adenocarcinoma), Hela (human cervical carcinoma), HL60 (human promyelocytic leukemia), and Huh-7 (human hepatocarcinoma), according to the reported CCK-8 method [23], which were purchased from Shanghai Cell Bank, Chinese Academy of Sciences. In brief, these cell lines were incubated in in RPMI or DMEM media with 10% FBS and 1% penicillin/streptomycin under a Thermo/Forma Scientific CO_2_ Water Jacketed Incubator with 5% CO_2_ in air at 37 °C. Then they were treated with various concentration of compounds or control at a density of 400–800 cells/well in 384-well plates. Cell viability assay was determined by CCK-8 assay. After 72 h incubation, CCK-8 reagent [2-(2-methoxy-4-nitrophenyl)-3-(4-nitrophenyl)-5-(2, 4-disulfophenyl)-2*H*-tetrazolium, monosodium salt (WST-8)] (Dojindo, Japan) was added, and absorbance was measured at 450 nm using Envision 2104 multi-label Reader (Perkin Elmer, Waltham, MA, USA). Dose response curves were plotted to determine the IC_50_ values using Prism 5.0 (GraphPad Software Inc., San Diego, CA, USA).

### 3.7. Anti-Tuberculosis Assay

Anti-tuberculosis was also tested refer to reported protocols [23]. Autoluminescent *Mycobacterium tuberculosis* H37Ra were inoculated in a 50 mL centrifuge tube containing 5 mL 7H9 broth (Becton Dickinson) with 0.1% Tween 80 and 10% OADC, and then incubated at 37 °C When the cultures reached an OD600 nm of 0.3-1.0, the culture was diluted and 50 μL diluted H37Ra were inoculated in sterile 384-well plates, the RLU of which should be between 10,000 and 50,000 and be recorded as the base luminescent Day0. The compounds (**3**−**16)** and the positive drug (isoniazid, Sigma) were added to the 384-well plates in triplicate by the Echo520 with the final concentration of 50 µM. The luminescent value was detected for the following three days. The data were analyzed with the Excel compared to the DMSO control to estimate the inhibitory activity of the compounds.

## 4. Conclusions

Cytochalasans have continuously aroused considerable interest from chemists and pharmacologists owing to their structural complexity and pharmacological significance. Sixteen structurally diverse chaetoglobosins, 10-(indol-3-yl)-[13]cytochalasans, including a new one, 6-*O*-methyl-chaetoglobosin Q (**1**), were encountered in the coral-associated fungus *C. globosum* C2F17. The X-ray crystal structures and absolute configurations of the previously reported chaetoglobosin E (**6**), penochalasin G (**12**), and armochaetoglobin G (**13**), are described here for the first time. Moreover, chaetoglobosins E (**6**) and Fex (**11**) showed significant cytotoxicity against a panel of cancer cell lines with the IC_50_ values ranging from 1.4 to 9.2 μM. Collectively, this work would expand the chemical space of the family of cytochalasans with potential antitumor significance.

## Figures and Tables

**Table 1:** The ^1^H (700 MHz), ^13^C (175 MHz) NMR and HMBC data of **1** (in DMSO-*d*_6_).

No.	δ_C_, Type	δ_H_ (*J* in Hz)	HMBC
1	173.6, C		
2-NH		8.13, s	1, 3, 4, 9
3	54.2, CH	3.44, m	1, 4, 5, 9, 3′
4	40.5, CH	2.91, dd (7.0, 2.1)	1, 3, 5, 6, 9, 10, 11, 23
5	37.4, CH	1.98, m	3, 4, 6, 7, 9, 11, 12
6	76.9, C		
7	71.9, CH	3.24, d (4.9)	6, 8, 9, 12, 13,
8	47.6, CH	2.27, t (10.5)	1, 7, 9,13, 23
9	61.9, C		
10	31.0, CH_2_	2.61, dd (14.7, 6.3) 2.51, overlapped	3, 4, 2′, 3′, 3a′
11	12.7, CH_3_	0.85, d (6.3)	4, 5, 6
12	19.5, CH_3_	1.13, s	5, 6, 7
13	129.5, CH	6.02, dd (14.0, 10.5)	8, 15
14	131.7, CH	4.93, m	8, 15, 16
15	41.8, CH_2_	2.24, m	13, 16, 17, 24,
16	31.6, CH	2.53, overlapped	
17	139.0, CH	5.45, d (9.1)	15, 16, 19, 24, 25
18	132.5, C		
19	81.8, CH	4.95, d (4.9)	17, 20, 21, 25
20	200.7, C		
21	132.5, CH	6.42, d (16.8)	19, 22, 23
22	136.0, CH	7.70, d (16.8)	20
23	199.3, C		
24 (16-Me)	20.9, CH_3_	0.95, d (6.3)	15, 16, 17
25 (18-Me)	10.8, CH_3_	1.31, s	17, 18, 19
26 (6-OMe)	49.2, CH_3_	3.04, s	6
1′-NH		10.86, s	2′, 3′, 1′a, 3′a
1′a	136.1, C		
2′	123.4, CH	7.08, d (2.1)	3′, 1′a, 3′a
3′	109.9, C		
3′a	127.3, C		
4′	118.1, CH	7.46, d (8.4)	3′, 6′, 1′a
5′	118.3, CH	6.95, t (7.7)	7′, 3′a
6′	120.9, CH	7.04, t (7.7)	4′, 1′a
7′	111.4, CH	7.31, d (8.4)	5′, 3′a
7-OH		4.13, d (8.4)	6, 7, 8
19-OH		5.22, d (4.9)	19, 18, 20

## Supplementary Materials

The following are available online at https://www.mdpi.com/1420-3049/25/5/1237/s1, the NMR, HR-ESIMS, UV, and IR spectra of compound **1** (Supplementary Figures S1–S10), the calculated ECD data of **1** (Supplementary Figure S11 and Tables S1–S2), physicochemical data of known compounds **2**‒**16** and the ITS sequence of *C. globosum* C2F17 are available online at http://www.mdpi.com/.
