# Supplementary material for: HPLC-DAD-Guided Isolation of Diversified Chaetoglobosins from the Coral-Associated Fungus Chaetomium globosum C2F17
Source: Molecules. 2020 Mar 9;25(5):1237. doi: 10.3390/molecules25051237 (PMC7179451; doi:10.3390/molecules25051237)
Supplement: Supplementary file 1 [file molecules-25-01237-s001.pdf]

Supplementary Materials

HPLC-DAD-Guided Isolation of Diversified Chaetoglobosins from the Coral-Associated Fungus *Chaetomium globosum* C2F17

Xiao-Wei Luo[1], Cheng-Hai Gao[1], Hu-Mu Lu[1], Jia-Min Wang[2], Zi-Qi Su[3], Hua-Ming Tao[3], Xue-Feng Zhou[2], Bin Yang[2*], and Yong-Hong Liu[1,2*]

[1] Institute of Marine Drugs, Guangxi University of Chinese Medicine, Nanning 530200, China; luoxiaowei1991@126.com (X.-W.L.); gaochh@gxtcmu.edu.cn (C.-H.G.); lhm098@126.com (H.-M. L.);

[2] CAS Key Laboratory of Tropical Marine Bio-resources and Ecology/Guangdong Key Laboratory of Marine Materia Medica, South China Sea Institute of Oceanology, Chinese Academy of Sciences, Guangzhou 510301, China; wjm2275322055@163.com (J.-M.W.); xfzhou@scsio.ac.cn (X.-F.Z.);

[3] School of Traditional Chinese Medicine, Southern Medical University, Guangzhou, China; ziqisl@163.com (Z.-Q.S.); taohm@smu.edu.cn (H.-M.T.)

* Correspondence: bingo525@163.com (B. Y.); yonghongliu@scsio.ac.cn (Y.-H. L.); Tel/Fax: +86-20-89023174 (B.Y.); +86-20-89023244 (Y.-H.L.).

Contents of Supporting Information

Figure S1. ^{1}H NMR spectrum of 6-*O*-methyl-chaetoglobosin Q (**1**) (DMSO-d_6, 700 MHz)..3
Figure S2. ^{13}C NMR spectrum of 6-*O*-methyl-chaetoglobosin Q (**1**) (DMSO-d_6, 175 MHz).4
Figure S3. DEPT 135 NMR spectrum of 6-*O*-methyl-chaetoglobosin Q (**1**) (DMSO-d_6, 175 MHz)........5
Figure S4. HSQC spectrum of 6-*O*-methyl-chaetoglobosin Q (**1**) (DMSO-d_6)........6
Figure S6. ^{1}H-^{1}H COSY spectrum of 6-*O*-methyl-chaetoglobosin Q (**1**) (DMSO-d_6)........8
Figure S7. NOESY spectrum of 6-*O*-methyl-chaetoglobosin Q (**1**) (DMSO-d_6)........9
Figure S8. HRESIMS spectrum of 6-*O*-methyl-chaetoglobosin Q (**1**)........10
Figure S9. UV spectrum of 6-*O*-methyl-chaetoglobosin Q (**1**)........11
Figure S10. IR spectrum of 6-*O*-methyl-chaetoglobosin Q (**1**)........11
Table S1. Energies of 6-*O*-methyl-chaetoglobosin Q (**1**) at MMFF94 force field........12
Table S2. Energies of 6-*O*-methyl-chaetoglobosin Q (**1**) at B3LYP/6–31+g(d) level in methanol........12
Figure S11. The optimized conformers and equilibrium populations of **1**. 13
Physicochemical data of known compounds **2–16**........13
Table S3. Cytotoxicity activities of compounds **3**–**16**. (IC_{50}, μM)........21
Figure S12. HPLC profiles of the EtOAc extracts of *Chaetomium globosum* C2F17........22
Figure S13. The ITS sequence of *Chaetomium globosum* C2F17 22

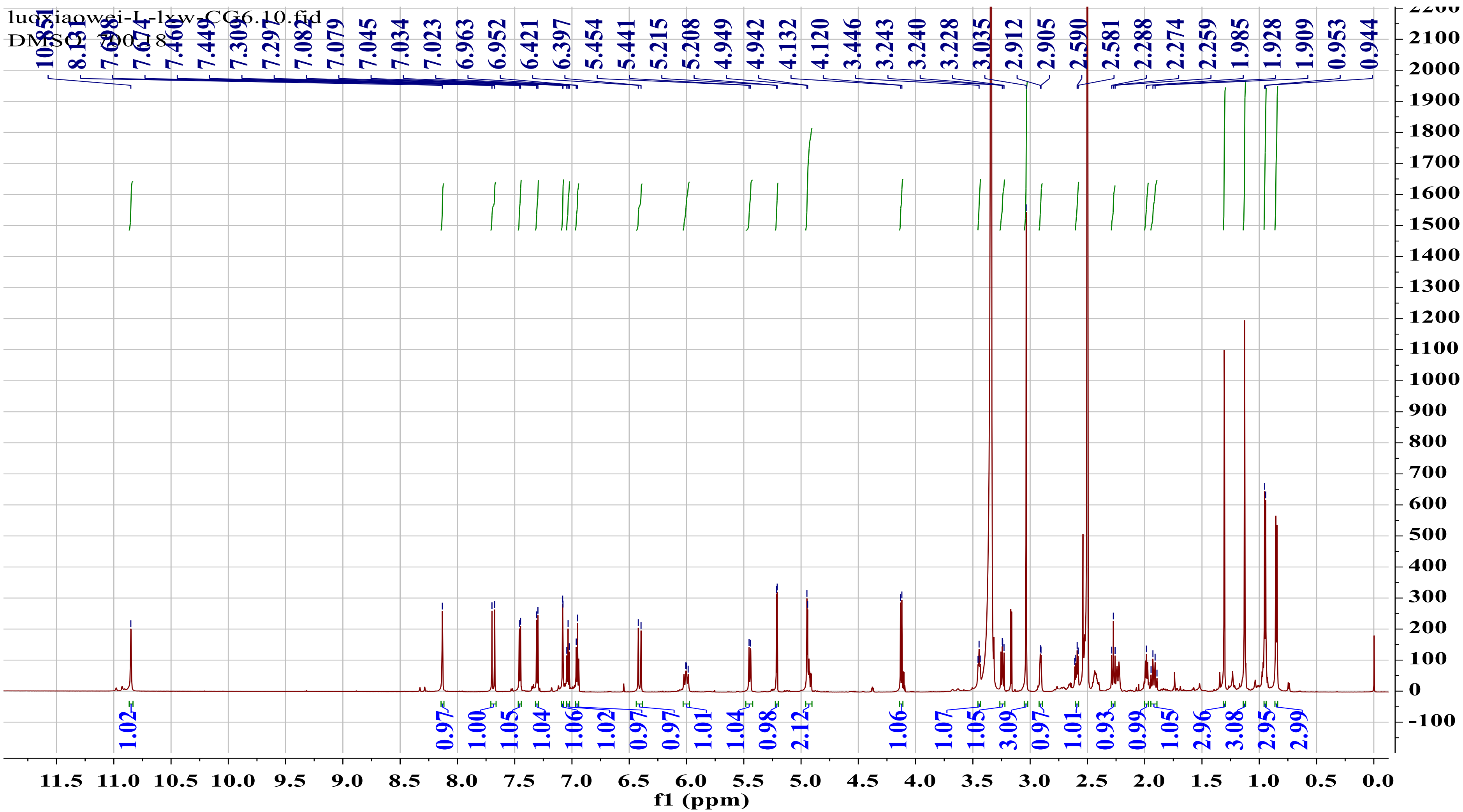

Figure S1. ^{1}H NMR spectrum of 6-*O*-methyl-chaetoglobosin Q (**1**) (DMSO-d_6, 700 MHz)

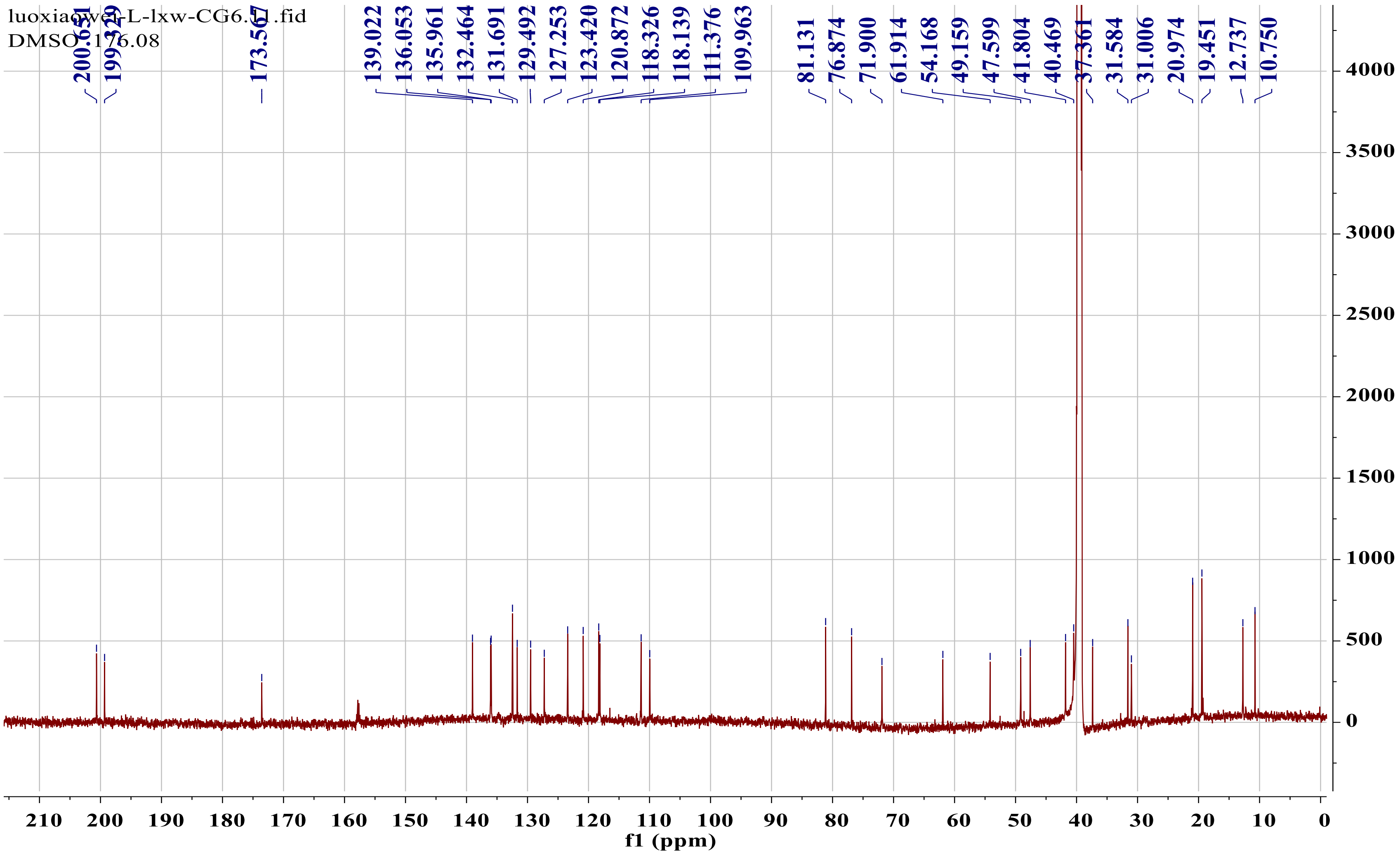

Figure S2. ^{13}C NMR spectrum of 6-*O*-methyl-chaetoglobosin Q (**1**) (DMSO-d_6, 175 MHz)

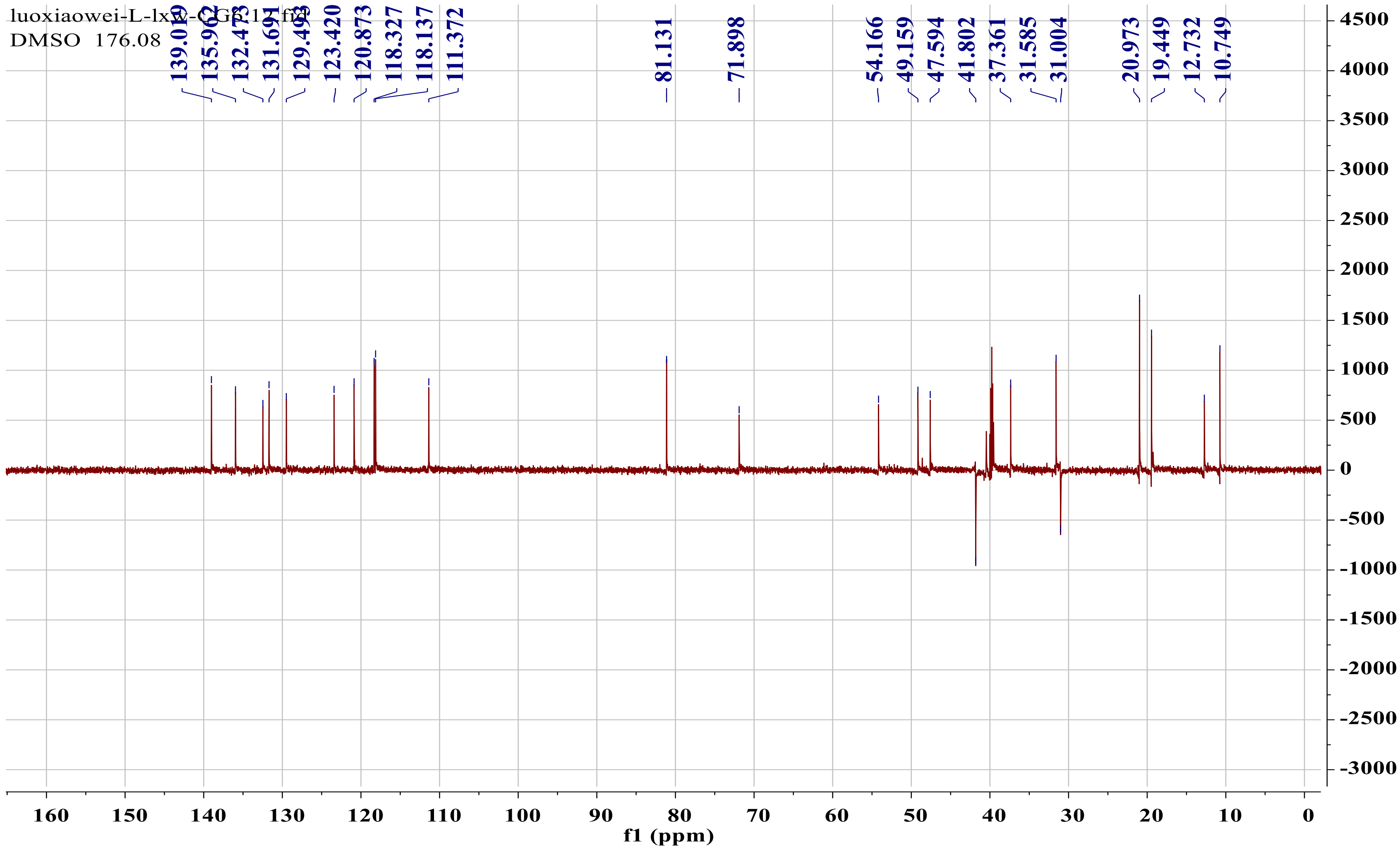

Figure S3. DEPT 135 NMR spectrum of 6-*O*-methyl-chaetoglobosin Q (**1**) (DMSO-d_6, 175 MHz)

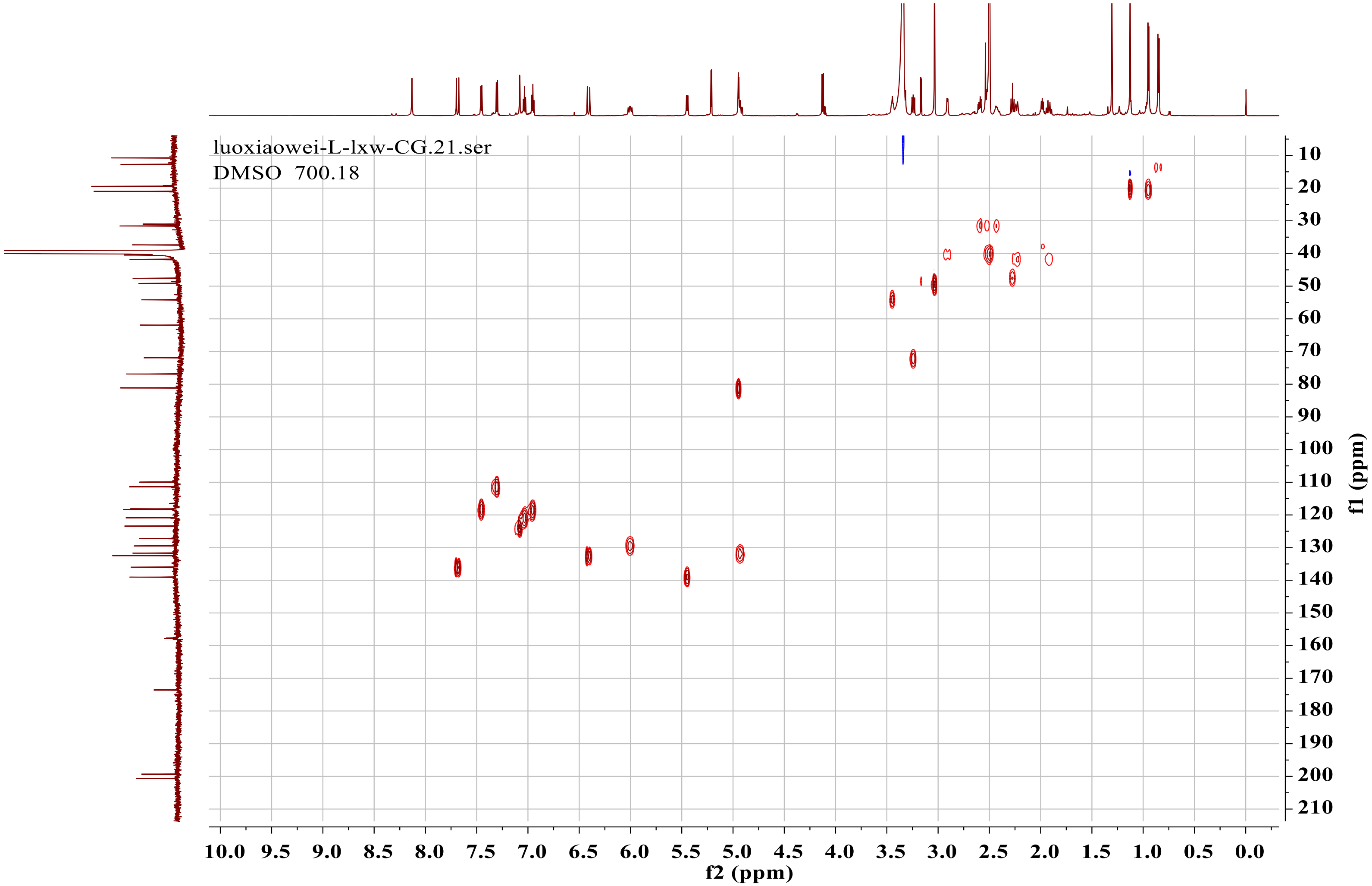

Figure S4. HSQC spectrum of 6-*O*-methyl-chaetoglobosin Q (**1**) (DMSO-d_6)

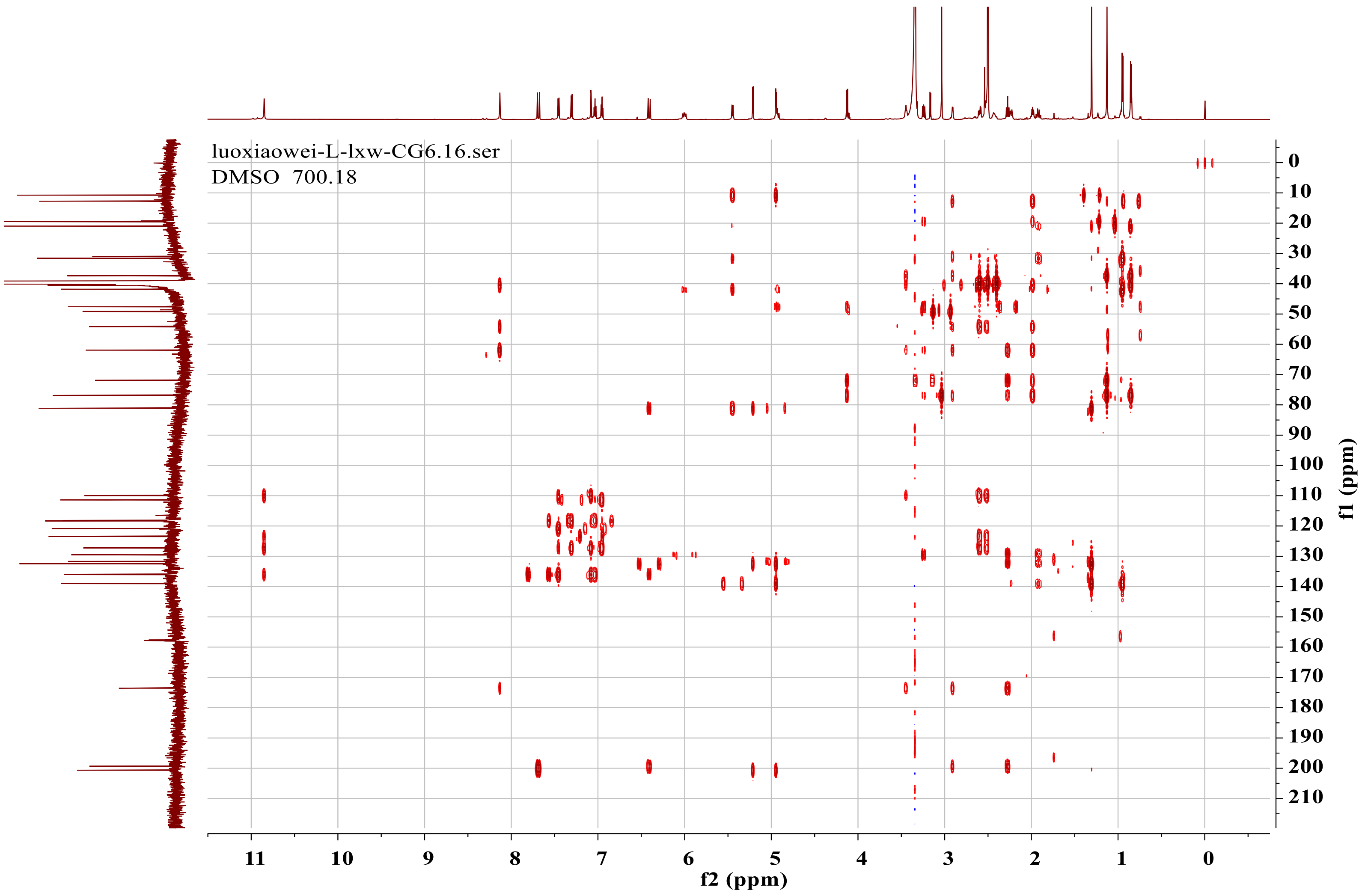

Figure S5. HMBC spectrum of 6-*O*-methyl-chaetoglobosin Q (**1**) (DMSO-*d*₆)

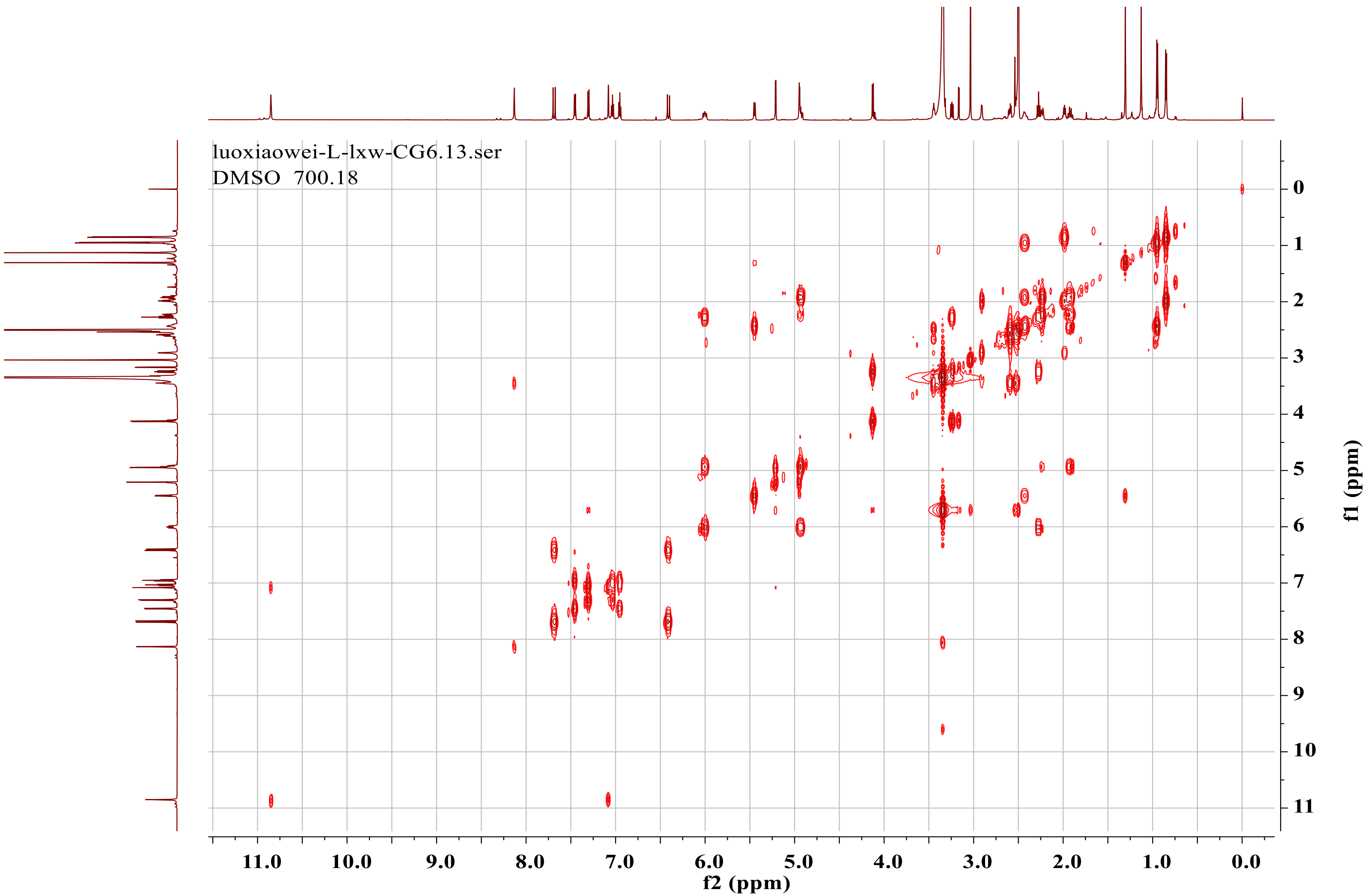

Figure S6. ^{1}H-^{1}H COSY spectrum of 6-*O*-methyl-chaetoglobosin Q (**1**) (DMSO-d_6)

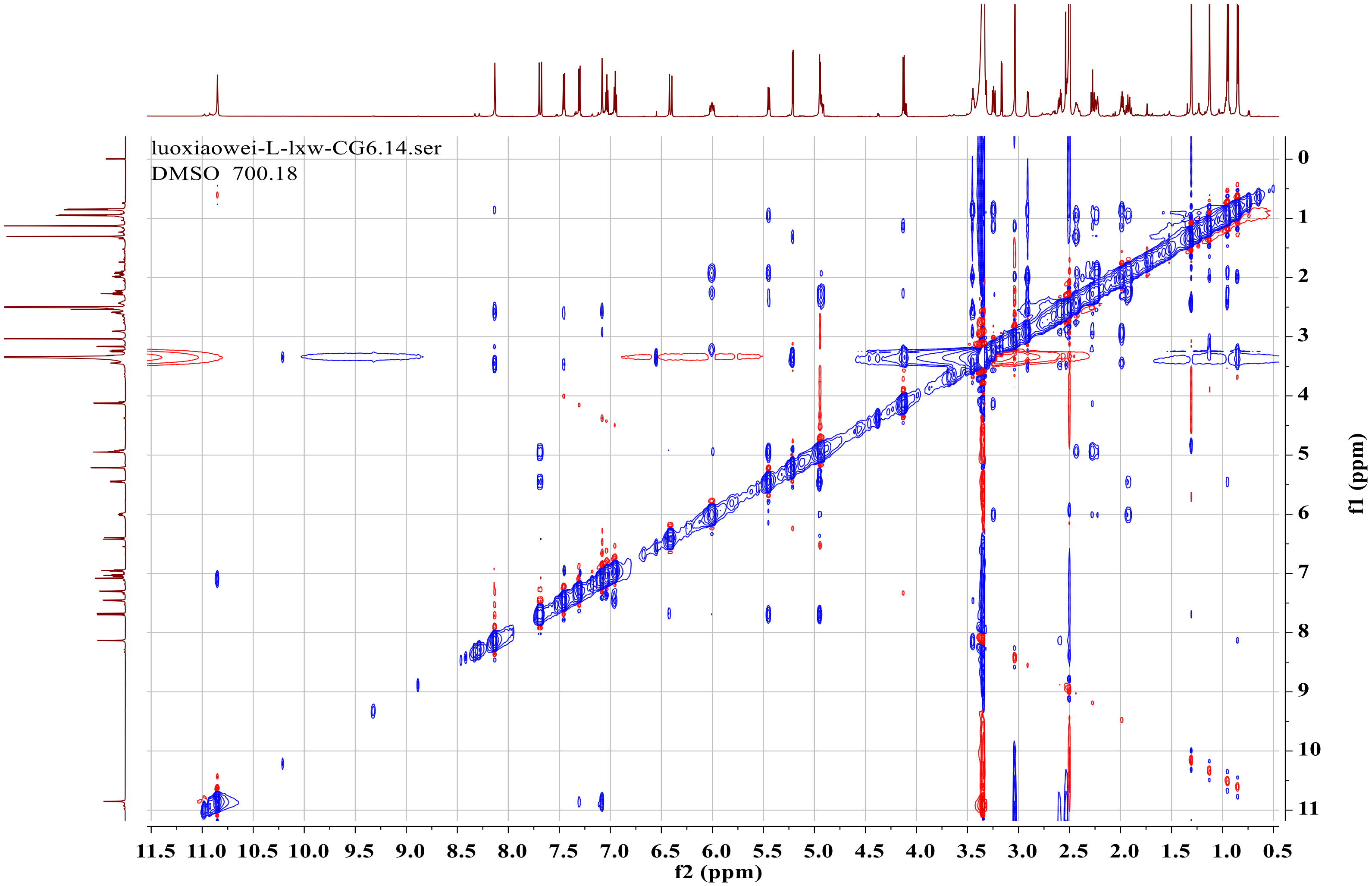

Figure S7. NOESY spectrum of 6-*O*-methyl-chaetoglobosin Q (**1**) (DMSO-d_6)

Mass Spectrum SmartFormula Report

Analysis Info		Acquisition Date	12/23/2019 9:15:28 AM	
Analysis Name	D:\Data\MS\data\201912\luoxiaowei_lxw-CG6_pos_61_01_7930.d			
Method	LC_Direct Infusion_pos_70-500mz.m	Operator	SCSIO	
Sample Name	luoxiaowei_lxw-CG6_pos	Instrument	maXis	255552.00029
Comment				

Acquisition Parameter

Source Type	ESI	Ion Polarity	Positive	Set Nebulizer	0.4 Bar
Focus	Active	Set Capillary	4500 V	Set Dry Heater	180 °C
Scan Begin	70 m/z	Set End Plate Offset	-500 V	Set Dry Gas	4.0 l/min
Scan End	1500 m/z	Set Charging Voltage	0 V	Set Divert Valve	Waste
		Set Corona	0 nA	Set APCI Heater	0 °C

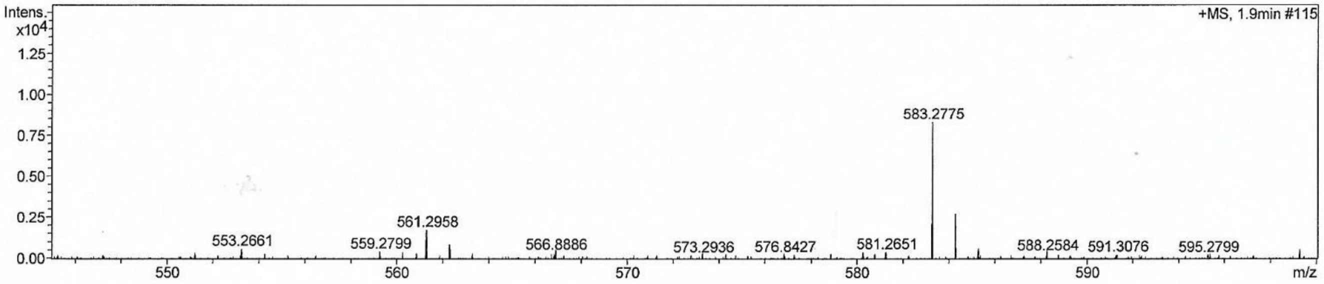

Meas. m/z	#	Ion Formula	Score	m/z	err [ppm]	err [mDa]	mSigma	rdb	e⁻ Conf	N-Rule
561.2958	1	C33H41N2O6	100.00	561.2959	0.2	0.1	57.6	14.5	even	ok
583.2775	1	C33H40N2NaO6	100.00	583.2779	0.6	0.3	21.4	14.5	even	ok
1121.5825	1	C66H81N4O12	100.00	1121.5846	-1.8	-2.0	59.1	28.5	even	ok
1143.5624	1	C66H80N4NaO12	100.00	1143.5665	-3.6	-4.1	125.8	28.5	even	ok

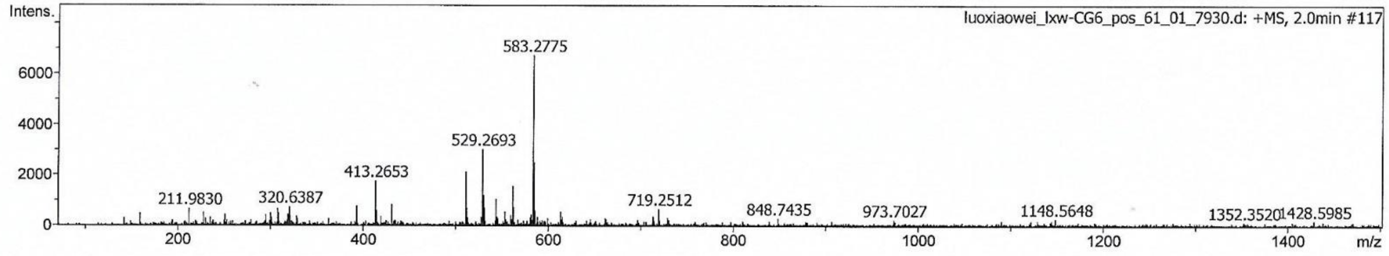

Figure S8. HRESIMS spectrum of 6-*O*-methyl-chaetoglobosin Q (**1**)

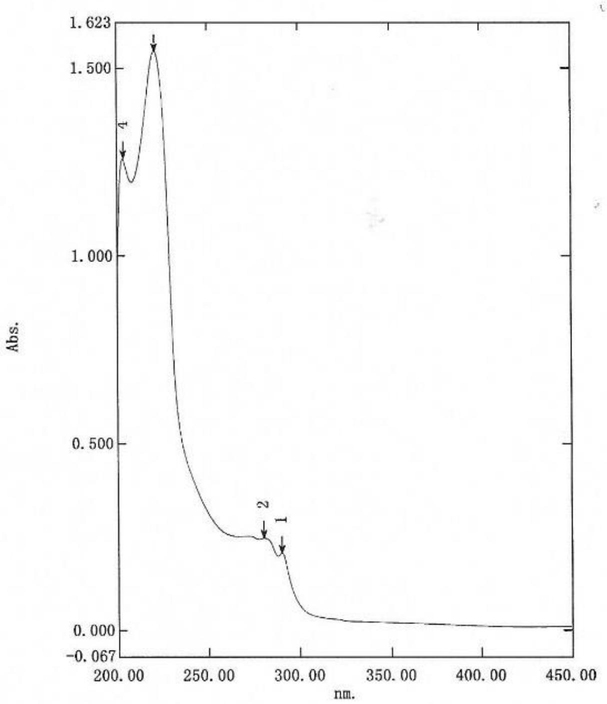

No.	P/V	波长(nm)	吸收值	描述
1	↑	290.00	0.207	3.37
2	↑	280.00	0.247	3.44
3	↑	221.20	1.546	4.24
4	↑	203.20	1.257	4.15

Figure S9. UV spectrum of 6-*O*-methyl-chaetoglobosin Q (**1**)

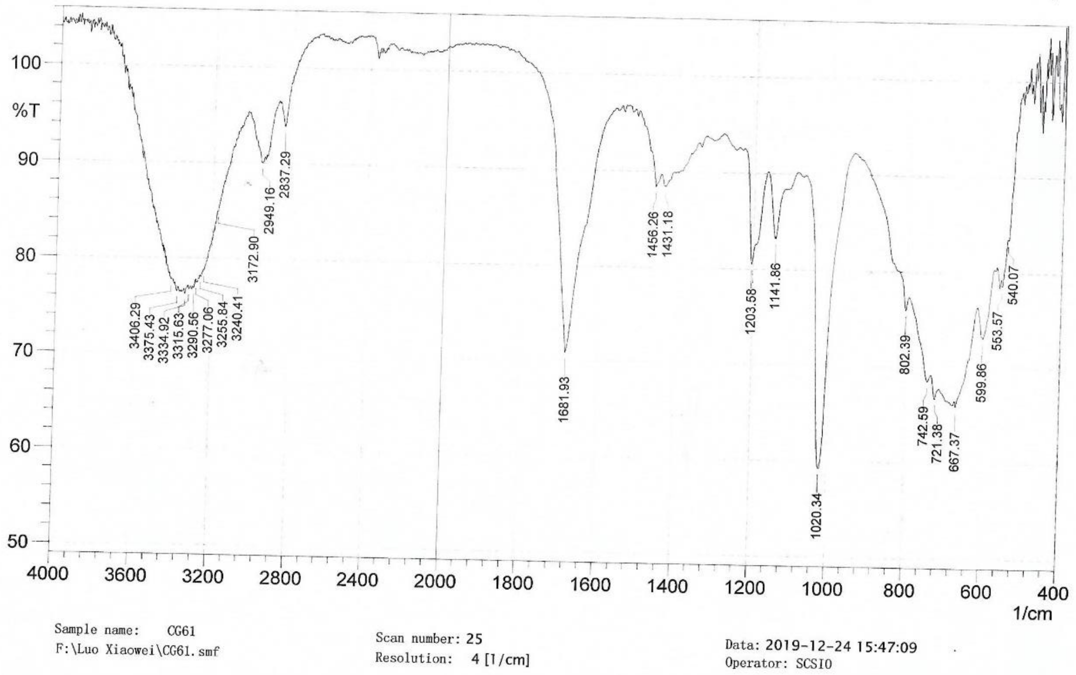

Figure S10. IR spectrum of 6-*O*-methyl-chaetoglobosin Q (**1**)

Table S1. Energies of 6-*O*-methyl-chaetoglobosin Q (**1**) at MMFF94 force field.

Conformer	Energy (kcal/mol)	Population (%)
1	592.16	40.1
2	593.35	24.9
3	595.94	8.7
4	596.11	8.2
5	597.41	4.8
6	598.36	3.3
7	598.70	2.9
8	599.92	1.8
9	600.43	1.4
10	601.02	1.1

Table S2. Energies of 6-*O*-methyl-chaetoglobosin Q (**1**) at B3LYP/6–31+g(d) level in methanol.

Conformer	E (Hartree)	E (kcal/mol)	Population (%)
1	–1842.911077	–1156445.12992827	6.4
2	–1842.9104809	–1156444.75586956	3.4
3	–1842.9096339	–1156444.22436859	1.39
4	–1842.910884	–1156445.00881884	5.21
5	–1842.9097034	–1156444.26798053	1.49
6	–1842.9107904	–1156444.9500839	4.72
7	–1842.9116116	–1156445.46539512	11.27
8	–1842.9124485	–1156445.99055823	27.37
9	–1842.9109031	–1156445.02080428	5.32
10	–1842.912637	–1156446.10884387	33.43

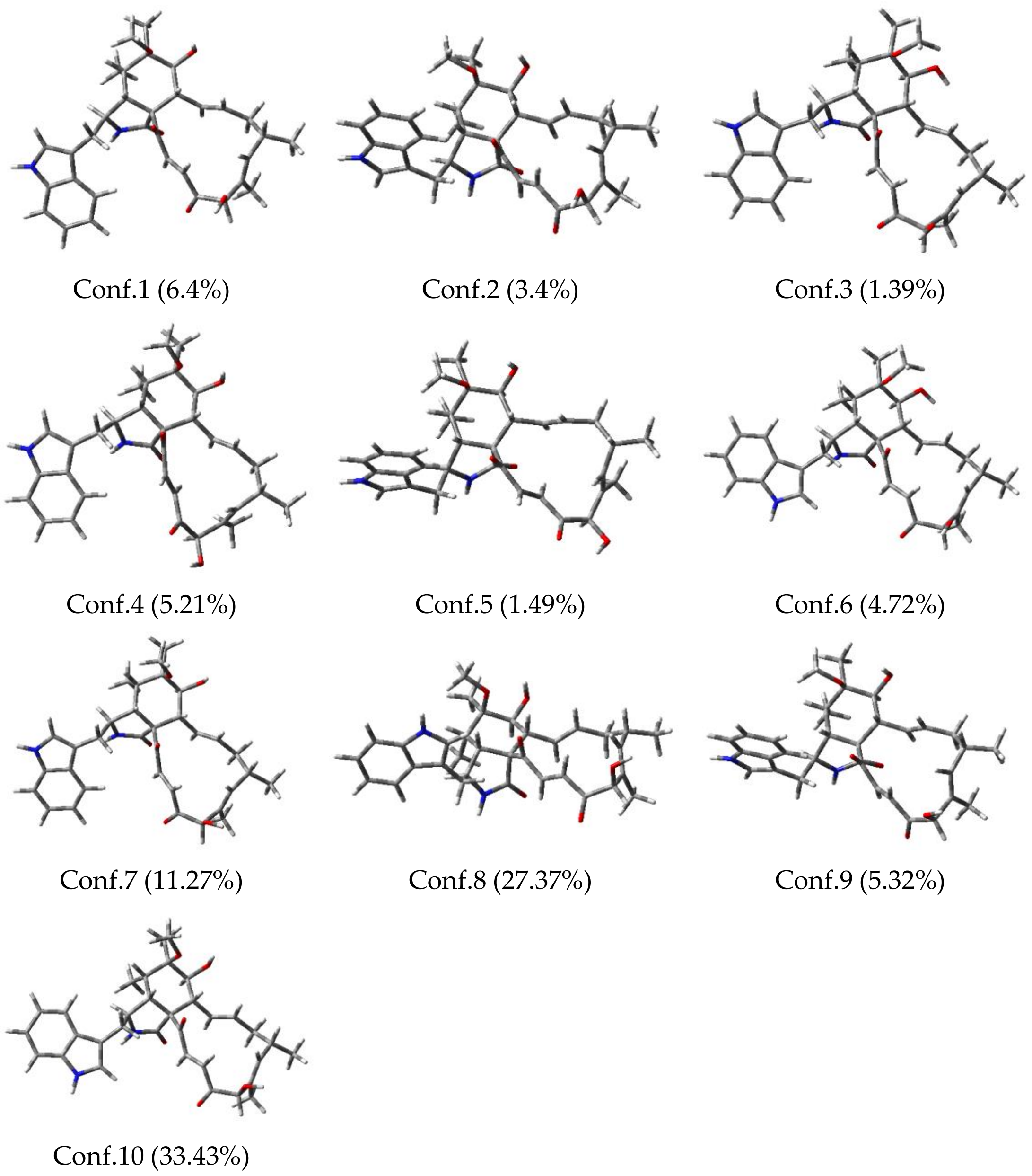

Figure S11. The optimized conformers and equilibrium populations of **1**.

Physicochemical data of known compounds **2–16**

Chaetoglobosin A (**2**): white amorphous powder; ^{1}H NMR (700 MHz, DMSO-d_6): δ_H 10.87 (1H, s, 1'-NH), 8.30 (1H, s, 2-NH), 7.37 (1H, d, *J* = 8.4 Hz, H-4'), 7.29 (1H, d, *J* = 8.4 Hz, H-7'), 7.25 (1H, d, *J* = 16.1 Hz, H-22), 7.08 (1H, d, *J* = 2.1 Hz, H-2'), 7.02 (1H, t, *J* = 7.7 Hz, H-6'), 6.94 (1H, t, *J* = 7.7 Hz, H-5'), 6.68 (1H, d, *J* = 16.1 Hz, H-21), 6.06 (1H, dd, *J* = 16.8, 10.5 Hz, H-13), 5.35 (1H, d, *J* = 9.8 Hz, H-17), 5.08 (1H, ddd, *J* = 14.0, 12.0, 2.1 Hz, H-14), 5.24 (1H, d, *J* = 2.7 Hz, OH-19), 4.90 (1H, br s, H-19), 3.65 (1H, m, H-3), 2.76 (1H, dd, *J* = 14.0, 3.5 Hz, H-10a), 2.67 (1H, d, *J* = 4.9 Hz, H-7), 2.45 (1H, dd, *J* = 14.0, 8.4 Hz, H-10b), 2.50 (1H, overlapped,

H-4), 2.50 (1H, overlapped, H-16), 2.24 (1H, overlapped, H-15b), 2.24 (1H, m, H-8), 1.85 (1H, dd, *J* = 24.5, 11.9 Hz, H-15a), 1.79 (1H, m, H-5), 1.34 (3H, s, H_3-25), 1.09 (3H, s, H_3-12), 0.95 (3H, d, *J* = 7.2 Hz, H_3-24), 0.65 (3H, d, *J* = 7.2 Hz, H_3-11); ^{13}C NMR (175 MHz, DMSO-d_6) δ_C: 200.4 (C-20), 199.5 (C-23), 172.7 (C-1), 137.5 (C-17), 136.1 (C-1'a), 136.0 (C-22), 133.8 (C-14), 133.5 (C-21), 131.6 (C-18), 127.4 (C-3'a), 127.1 (C-13), 124.1 (C-5'), 120.8 (C-2'), 118.5 (C-6'), 118.3 (C-4'), 111.4 (C-7'), 109.2 (C-3'), 81.6 (C-19), 63.9 (C-9), 61.3 (C-7), 57.1 (C-6), 52.2 (C-3), 45.0 (C-4), 45.9 (C-8), 40.1 (C-15), 35.3 (C-5), 32.7 (C-10), 31.8 (C-16), 20.0 (C-24), 19.2 (C-12), 12.2 (C-11), 10.6 (C-25).

Chaetoglobosin B (**3**): white amorphous powder; ^{1}H NMR (700 MHz, DMSO-d_6): δ_H 10.92 (1H, s, 1'-NH), 8.09 (1H, s, 2-NH), 7.40 (1H, d, *J* = 7.7 Hz, H-4'), 7.35 (1H, d, *J* = 8.4 Hz, H-7'), 7.33 (1H, d, *J* = 16.8 Hz, H-22), 7.09 (1H, d, *J* = 2.1 Hz, H-2'), 7.06 (1H, t, *J* = 7.7 Hz, H-6'), 6.97 (1H, t, *J* = 7.7 Hz, H-5'), 6.92 (1H, d, *J* = 16.8 Hz, H-21), 5.93 (1H, dd, *J* = 15.4, 9.8 Hz, H-13), 5.43 (1H, d, *J* = 9.8 Hz, H-17), 5.33 (1H, d, *J* = 4.2 Hz, H-19), 5.00 (1H, d, *J* = 4.2 Hz, 19-OH), 4.99 (1H, overlapped, H-14), 4.59 (1H, d, *J* = 7.0 Hz, 7-OH), 3.66 (1H, t, *J* = 9.8 Hz, H-7), 3.41 (1H, overlapped, H-3), 2.97 (1H, br s, H-4), 2.74 (1H, dd, *J* = 14.0, 2.8 Hz, H-10a), 2.53 (1H, m, H-16), 2.48 (1H, dd, *J* = 14.0, 9.1 Hz, H-10b), 2.27 (1H, m, H-8), 2.03 (1H, m, H-15a), 1.84 (1H, m, H-15b), 1.19 (3H, s, H_3-25), 1.38 (3H, s, H_3-11), 1.54 (3H, s, H_3-12), 0.96 (3H, d, *J* = 7.2 Hz, H_3-24) ; ^{13}C NMR (175 MHz, DMSO-d_6) δ_C: 200.3 (C-23), 200.1 (C-20), 172.6 (C-1), 138.0 (C-17), 136.7 (C-22), 136.2 (C-1'a), 134.4 (C-21), 133.8 (C-6), 133.4 (C-14), 131.4 (C-18), 127.2 (C-3'a), 126.7 (C-13), 125.3 (C-5), 123.6 (C-2'), 121.0 (C-6'), 118.5 (C-5'), 118.0 (C-4'), 111.6 (C-7'), 109.9 (C-3'), 82.0 (C-19), 67.7 (C-7), 60.7 (C-9), 57.8 (C-4), 50.0 (C-3), 47.1 (C-8), 40.3 (C-15), 32.0 (C-16), 31.7 (C-10), 21.1 (C-24), 17.0 (C-12), 14.7 (C-11), 10.7 (C-25).

Chaetoglobosin C (**4**): white amorphous powder; ^{1}H NMR (700 MHz, DMSO-d_6): δ_H 10.96 (1H, s, 1'-NH), 8.48 (1H, s, 2-NH), 7.55 (1H, d, *J* = 8.4 Hz, H-4'), 7.33 (1H, d, *J* = 8.4 Hz, H-7'), 7.14 (1H, d, *J* = 2.1 Hz, H-2'), 7.07 (1H, t, *J* = 7.7 Hz, H-6'), 7.05 (1H, t, *J* = 7.7 Hz, H-5'), 6.08 (1H, dd, *J* = 14.7, 9.8 Hz, H-13), 5.90 (1H, d, *J* = 9.8 Hz, H-17), 4.95 (1H, ddd, *J* = 14.0, 12.0, 2.1 Hz, H-14), 3.76 (1H, m, H-3), 2.85 (1H, dd, *J* = 14.7, 5.6 Hz, H-10a), 2.72 (1H, dd, *J* = 14.0, 3.5 Hz, H-10b), 2.68 (H, m, H-22a), 2.64 (1H, d, *J* = 5.6 Hz, H-7), 2.61 (1H, m, H-22a),

2.42 (1H, m, H-16), 2.30 (1H, m, H-22b), 2.25 (1H, m, H-4), 2.14 (1H, dd, *J* = 9.8, 5.6 Hz, H-8), 1.84 (1H, dd, *J* = 24.5, 11.9 Hz, H-15a), 1.78 (1H, m, H-5), 1.71 (1H, m, H-21a), 1.68 (3H, s, H_3-25), 1.61 (1H, m, H-15b), 1.56 (1H, m, H-21b), 1.17 (3H, s, H_3-12), 0.95 (3H, d, *J* = 7.2 Hz, H_3-24), 0.93 (3H, d, *J* = 7.2 Hz, H_3-11); ^{13}C NMR (175 MHz, DMSO-d_6) δ_C: 208.2 (C-19), 205.4 (C-20), 196.2 (C-23), 174.0 (C-1), 155.9 (C-17), 135.9 (C-1'a), 133.3 (C-14), 131.1 (C-18), 127.8 (C-3'a), 127.1 (C-13), 125.3 (C-5'), 121.2 (C-2'), 118.8 (C-6'), 118.4 (C-4'), 111.4 (C-7'), 108.1 (C-3'), 62.3 (C-9), 60.4 (C-7), 56.8 (C-6), 52.4 (C-3), 48.5 (C-4), 48.3 (C-8), 39.2 (C-15), 37.2 (C-22), 36.2 (C-5), 32.6 (C-16), 32.0 (C-10), 31.8 (C-21), 19.4 (C-24), 19.2 (C-12), 12.4 (C-11), 10.1 (C-25).

Chaetoglobosin D (**5**): white amorphous powder; ^{1}H NMR (700 MHz, DMSO-d_6): δ_H 10.85 (1H, s, 1'-NH), 8.03 (1H, s, 2-NH), 7.43 (1H, d, *J* = 16.1 Hz, H-22), 7.37 (1H, d, *J* = 7.7 Hz, H-4'), 7.29 (1H, d, *J* = 8.4 Hz, H-7'), 7.06 (1H, d, *J* = 2.1 Hz, H-2'), 7.01 (1H, t, *J* = 7.7 Hz, H-5'), 6.94 (1H, t, *J* = 7.7 Hz, H-6'), 6.55 (1H, d, *J* = 16.5 Hz, H-21), 5.83 (1H, dd, *J* = 16.1, 9.8 Hz, H-13), 5.39 (1H, d, *J* = 9.1 Hz, H-17), 5.24 (1H, d, *J* = 4.9 Hz, H-12a), 5.00 (1H, ddd, *J* = 16.1, 11.0, 9.8 Hz, H-14), 4.92 (1H, d, *J* = 4.9 Hz, H-12b), 4.85 (1H, br s, H-19), 4.79 (1H, d, *J* = 6.3 Hz, 7-OH), 3.68 (1H, dd, *J* = 9.8, 6.3 Hz, H-7), 3.29 (1H, m, H-3), 3.17 (1H, d, *J* = 4.9 Hz, H-4), 2.70 (1H, m, H-10a), 2.56 (3H, m, H-10b, H-5, H-8), 2.36 (1H, m, H-16), 1.99 (1H, dd, *J* = 24.5, 11.2 Hz, H-15b), 1.84 (1H, dd, *J* = 24.5, 11.2 Hz, H-15a), 1.34 (3H, s, H_3-25), 0.94 (3H, d, *J* = 7.0 Hz, H_3-24), 0.67 (3H, d, *J* = 7.0 Hz, H_3-11); ^{13}C NMR (175 MHz, DMSO-d_6) δ_C: 200.38 (C-20), 199.4 (C-23), 171.2 (C-1), 151.0 (C-6), 138.0 (C-17), 136.1 (C-1'a), 134.7 (C-21), 134.5 (C-14), 133.4 (C-22), 131.7 (C-18), 127.5 (C-13), 127.4 (C-3'a), 124.1 (C-5'), 120.8 (C-2'), 118.4 (C-6'), 118.0 (C-4'), 111.8 (C-12), 111.4 (C-7'), 109.3 (C-3'), 81.5 (C-19), 70.2 (C-7), 61.2 (C-9), 51.8 (C-3), 47.8 (C-8), 44.8 (C-4), 40.7 (C-15), 32.3 (C-16), 31.8 (C-5), 31.1 (C-10), 21.1 (C-24), 13.2 (C-11), 10.6 (C-25).

Chaetoglobosin E (**6**): white amorphous powder; ^{1}H NMR (700 MHz, DMSO-d_6): δ_H 10.92 (1H, s, 1'-NH), 8.10 (1H, s, 2-NH), 7.40 (1H, d, *J* = 7.7 Hz, H-4'), 7.35 (1H, d, *J* = 8.4 Hz, H-7'), 7.06 (1H, d, *J* = 2.1 Hz, H-2'), 7.07 (1H, t, *J* = 7.7 Hz, H-6'), 6.98 (1H, t, *J* = 7.7 Hz, H-5'), 6.22 (1H, dd, *J* = 14.7, 9.8 Hz, H-13), 6.29 (1H, d, *J* = 9.8 Hz, H-17), 5.06 (1H, m, H-14), 4.88 (1H, d, *J* = 5.6 Hz, 20-OH), 4.77 (1H, m, H-20), 4.52 (1H, d, *J* = 7.7 Hz, 7-OH), 3.61 (1H, m,

H-3), 2.94 (1H, dt, *J* = 19.6, 7.2 Hz, H-22a), 2.79 (1H, s, H-4), 2.73 (3H, overlappped, H-10a, H-16, H-22b), 2.36 (2H, m, H-15a, H-10b), 2.00 (2H, m, H-8, H-15b), 1.76 (2H, m, H_2-21), 1.71 (3H, s, H_3-25), 1.51 (3H, s, H_3-11), 1.11 (3H, s, H_3-12), 1.00 (3H, d, *J* = 7.2 Hz, H_3-24) ; ^{13}C NMR (175 MHz, DMSO-d_6) δ_C: 210.2 (C-23), 204.3 (C-19), 173.9 (C-1), 148.2 (C-17), 136.2 (C-1'a), 135.0 (C-18), 133.5 (C-6), 133.4 (C-14), 128.5 (C-13), 127.1 (C-3'a), 125.5 (C-5), 123.6 (C-2'), 121.0 (C-6'), 118.4 (C-5'), 117.9 (C-4'), 111.6 (C-7'), 109.9 (C-3'), 70.1 (C-20), 67.6 (C-9), 61.6 (C-7), 57.1 (C-3), 51.0 (C-8), 49.3 (C-4), 40.7 (C-15), 36.4 (C-22), 32.9 (C-16), 31.8 (C-10), 30.5 (C-21), 19.8 (C-24), 16.9 (C-12), 14.7 (C-11), 12.0 (C-25).

Chaetoglobosin F (**7**): white amorphous powder; ^{1}H NMR (700 MHz, DMSO-d_6): δ_H 10.90 (1H, s, 1'-NH), 8.32 (1H, s, 2-NH), 7.44 (1H, d, *J* = 7.7 Hz, H-4'), 7.33 (1H, d, *J* = 8.4 Hz, H-7'), 7.09 (1H, d, *J* = 2.1 Hz, H-2'), 7.05 (1H, t, *J* = 7.7 Hz, H-6'), 6.97 (1H, t, *J* = 7.7 Hz, H-5'), 6.33 (1H, dd, *J* = 14.7, 9.8 Hz, H-13), 6.19(1H, d, *J* = 9.8 Hz, H-17), 5.16 (1H, m, H-14), 4.82 (1H, d, *J* = 5.6 Hz, 20-OH), 4.58 (1H, m, H-20), 3.64 (1H, m, H-3), 2.75 (1H, dd, *J* = 14.0, 3.5 Hz, H-10a), 2.71 (1H, m, H-16), 2.65 (1H, d, *J* = 5.6 Hz, H-7), 2.58 (2H, m, H-10b, H-22a), 2.34 (2H, m, H-4, H-15a), 2.19 (1H, dd, *J* = 9.8, 5.6 Hz, H-8), 2.12(1H, m, H-22b), 1.98 (1H, dd, *J* = 24. 5, 11. 9 Hz, H-15b), 1.69 (3H, s, H_3-25), 1.58 (3H, m, H-5, H-21a, H-21b), 1.09 (3H, s, H_3-12), 0.98 (3H, d, *J* = 7.2 Hz, H_3-24), 0.61 (3H, d, *J* = 7.2 Hz, H_3-11); ^{13}C NMR (175 MHz, DMSO-d_6) δ_C: 209.2 (C-23), 204.1(C-19), 173.7 (C-1), 147.8 (C-17), 136.1 (C-1'a), 135.0 (C-18), 132.9 (C-14), 128.5 (C-13), 127.6 (C-3'a), 124.4 (C-2'), 120.9 (C-6'), 118.5 (C-5'), 118.0 (C-4'), 111.5 (C-7'), 109.0 (C-3'), 70.3 (C-20), 63.7 (C-9), 61.3 (C-7), 57.0 (C-6), 52.2 (C-3), 47.9 (C-4), 46.9 (C-8), 40.2 (C-15), 36.5 (C-22), 35.7 (C-5), 32.9 (C-16), 32.8 (C-10), 30.0 (C-21), 19.7 (C-24), 19.2 (C-12), 12.2 (C-11), 12.1 (C-25).

Chaetoglobosin G (**8**): white amorphous powder; ^{1}H NMR (700 MHz, DMSO-d_6): δ_H 10.93 (1H, s, 1'-NH), 8.27 (1H, s, 2-NH), 7.43 (1H, d, *J* = 7.7 Hz, H-4'), 7.35 (1H, d, *J* =7.7 Hz, H-7'), 7.09 (1H, d, *J* = 2.1 Hz, H-2'), 7.07 (1H, t, *J* = 7.7 Hz, H-6'), 7.00 (1H, t, *J* = 7.7 Hz, H-5'), 6.13 (1H, dd, *J* = 16.1, 9.8 Hz, H-13), 6.06 (1H, d, *J* = 9.1 Hz, H-17), 4.94 (1H, ddd, *J* = 16.1, 11.0, 9.8 Hz, H-14), 4.46 (1H, d, *J* = 7.7 Hz, H-7), 3.58 (1H, m, H-3), 3.12 (1H, m, H-4), 2.84-3.05 (2H, m, H_2-10), 2.76 (2H, m, H-22), 2.44 (1H, m, H-16), 2.31-2.37 (2H, m, H_2-21), 2.31 (1H, m, H-15b), 2.02 (1H, t, *J* = 7.7 Hz, H-8), 1.94 (1H, m, H-15a), 1.74 (3H, s, H_3-12), 1.52 (3H, s,

H_3-11), 1.15 (3H, s, H_3-25), 1.00 (3H, d, *J* = 7.0 Hz, H_3-24); ^{13}C NMR (175 MHz, DMSO-*d*6) δ_C: 209.0 (C-23), 206.2 (C-20), 197.0 (C-19), 174.1 (C-1), 156.8 (C-17), 136.1 (C-1'a), 133.7 (C-6), 133.5 (C-14), 131.5 (C-18), 127.7 (C-13), 127.0 (C-3'a), 125.7 (C-5), 124.0 (C-5'), 121.0 (C-2'), 118.5 (C-6'), 118.0 (C-4'), 111.5 (C-7'), 109.4 (C-3'), 67.3 (C-7), 60.9 (C-9), 57.5 (C-4), 52.2 (C-3), 50.2 (C-8), 40.4 (C-15), 37.1 (C-22), 32.9 (C-16), 32.4 (C-21), 31.9 (C-10), 19.3 (C-24), 16.9 (C-12), 14.6 (C-11), 10.0 (C-25).

Aureochaeglobosin B (**9**): white amorphous powder; $[\alpha]_D^{25}$ −37.8 (*c* 0.2, MeOH); CD (0.2 mg/mL, MeOH): λ_{max} ($\Delta\varepsilon$) 204 (–10.3), 213 (−4.72), 219 (−5.7), 233 (+7.7), 306 (−2.6); ^{1}H NMR (500 MHz, $CDCl_3$): δ_H 7.61 (1H, d, *J* = 7.7 Hz, H-4'), 7.37 (1H, d, *J* =7.7 Hz, H-7'), 7.05 (1H, d, *J* = 2.1 Hz, H-2'), 7.14 (1H, t, *J* = 7.7 Hz, H-6'), 7.21 (1H, t, *J* = 7.7 Hz, H-5'), 6.54 (1H, dd, *J* = 16.1, 9.8 Hz, H-13), 6.10 (1H, s, H-35), 6.07 (1H, m, H-28), 6.02 (1H, s, H-34), 5.74 (1H, m, H-27), 5.68 (1H, s, H-36), 5.53 (1H, m, H-14), 5.39 (1H, dd, *J* = 9.0, 6.0 Hz, H-33), 5.33 (1H, d, *J* = 9.1 Hz, H-17), 4.66 (1H, s, H-19), 3.98 (1H, t, *J* = 8.5 Hz, H-38), 3.88 (1H, t, *J* = 5.0 Hz, H-21), 3.82 (2H, m, H-7, 31), 3.64 (1H, m, H-30), 3.59 (2H, m, H-22, 38b), 3.42 (1H, m, H-3), 2.88 (1H, m, H-29), 2.79 (1H, m, H-4), 2.76 (2H, m, H_2-10), 2.73 (1H, m, H-32), 2.67 (1H, m, H-16), 2.52 (2H, m, H-8, 26a), 2.36 (1H, m, H-15b), 2.35 (1H, m, H-26b), 2.03 (1H, m, H-15a), 1.74 (3H, s, H_3-12), 1.74 (3H, d, *J* = 5.5 Hz, H_3-37), 1.56 (3H, s, H_3-11), 1.46 (3H, s, H_3-25), 1.01 (3H, d, *J* = 7.0 Hz, H_3-24); ^{13}C NMR (125 MHz, $CDCl_3$) δ_C: 210.9 (C-20), 207.9 (C-23), 174.4 (C-1), 139.3 (C-14), 138.6 (C-17), 136.4 (C-1'a), 134.0 (C-6), 133.6 (C-35), 132.4 (C-18), 130.8 (C-34), 129.9 (C-36), 129.5 (C-28), 127.9 (C-33), 127.1 (C-3'a), 126.5 (C-13), 125.4 (C-5), 122.9 (C-27), 122.7 (C-5'), 122.4 (C-2'), 119.8 (C-7'), 118.7 (C-4'), 111.4 (C-6'), 111.7 (C-3'), 83.9 (C-19), 81.5 (C-30), 80.5 (C-31), 70.2 (C-38), 67.6 (C-7), 61.3 (C-9), 52.4 (C-32), 49.4 (C-4), 59.0 (C-3), 50.8 (C-8), 44.9 (C-22), 43.0 (C-21), 40.5 (C-15), 38.4 (C-29), 33.2 (C-16), 32.9 (C-10), 24.9 (C-26), 21.2 (C-24), 18.1 (C-37), 14.2 (C-12), 17.9 (C-11), 11.3 (C-25). HR-ESIMS *m/z* 735.4008 (calcd for $C_{45}H_{55}N_2O_7$, 735.4004), 757.3820 (calcd for $C_{45}H_{54}N_2NaO_7$, 757.3823).

Isochaetoglobosin D (**10**): white amorphous powder; ^{1}H NMR (700 MHz, DMSO-*d*6): δ_H 10.97 (1H, s, 1'-NH), 8.28 (1H, s, 2-NH), 7.55 (1H, d, *J* = 7.7 Hz, H-4'), 7.33 (1H, d, *J* = 8.4 Hz, H-7'), 7.14 (1H, d, *J* = 2.1 Hz, H-2'), 7.07 (1H, t, *J* = 7.7 Hz, H-6'), 7.00 (1H, t, *J* = 7.7 Hz, H-5'), 5.86 (1H, d, *J* = 9.1 Hz, H-17), 5.79 (1H, dd, *J* = 16.1, 9.8 Hz, H-13), 5.18 (1H, br s, H-12a),

4.96 (1H, br s, H-12b), 4.86 (1H, ddd, *J* = 16.1, 11.0, 9.8 Hz, H-14), 4.72 (1H, d, *J* = 5.6 Hz, 7-OH), 3.57 (1H, dd, *J* = 9.8, 6.3 Hz, H-7), 3.29 (1H, m, H-3), 2.89 (1H, dd, *J* = 14.7, 5.6 Hz, H-10a), 2.66 (2H, m, H-5, H-16), 2.62 (1H, dd, *J* = 14.7, 3.5 Hz, H-10b), 2.58 (1H, t, *J* = 10.5 Hz, H-8), 2.44 (1H, t, *J* = 10.5 Hz, H-21a), 2.31 (1H, dd, *J* = 18.2, 11.4 Hz, H-22a), 2.25 (1H, m, H-15a), 2.21 (1H, t, *J* = 4.2 Hz, H-4), 1.74 (1H, dd, *J* = 24.6, 11.4 Hz, H-15b), 1.57 (1H, dd, *J* = 10.5, 6.4 Hz, H-21b), 1.21 (1H, dd, *J* = 18.2, 7.8 Hz, H-22b), 1.69 (3H, s, H_3-25), 0.96 (3H, d, *J* = 7.0 Hz, H_3-11), 0.94 (3H, d, *J* = 7.0 Hz, H_3-24); ^{13}C NMR (175 MHz, DMSO-d_6) δ_C: 208.1 (C-23), 205.3 (C-20), 196.1 (C-19), 173.8 (C-1), 155.9 (C-17), 150.8 (C-6), 135.8 (C-1'a), 133.2 (C-14), 130.9 (C-3'a), 127.8 (C-18), 127.3 (C-13), 125.3 (C-5'), 121.0 (C-2'), 118.8 (C-6'), 118.5 (C-4'), 112.7 (C-12), 111.3 (C-7'), 108.4 (C-3'), 68.7 (C-7), 60.8 (C-9), 52.2 (C-3), 48.8 (C-8), 45.8 (C-4), 40.0 (C-15), 36.8 (C-22), 32.7 (C-16), 32.0 (C-5), 32.1(C-10), 30.6 (C-21), 19.2 (C-24), 13.4 (C-11), 10.1 (C-25).

Chaetoglobosin Fex (**11**): white amorphous powder; ^{1}H-NMR (700 MHz, DMSO-d_6): δ_H 10.90 (1H, s, 1'-NH), 8.05 (1H, s, 2-NH), 7.45 (1H, d, *J* = 7.8 Hz, H-4'), 7.36 (1H, d, *J* = 7.8 Hz, H-7'), 7.08 (1H, d, *J* = 2.4 Hz, H-2'), 7.08 (1H, t, *J* = 7.8 Hz, H-6'), 6.99 (1H, t, *J* = 7.8 Hz, H-5'), 6.16 (1H, d, *J* = 9.6 Hz, H-17), 6.08 (1H, dd, *J* = 15.0, 9.6 Hz, H-13), 5.06 (1H, s, H-12a), 5.04 (1H, m, H-14), 4.85 (1H, s, H-12b), 4.74 (1H, d, *J* = 6.6 Hz, 20-OH), 4.80 (1H, d, *J* = 7.8Hz, 7-OH), 4.52 (1H, m, H-20), 3.60 (1H, dd, *J* = 9.6, 6.0 Hz, H-7), 3.22 (1H, m, H-3), 2.68 (1H, m, H-16), 2.67 (1H, dd, *J* = 14.4, 4.2 Hz, H-10a), 2.61 (1H, dd, *J* = 14.4, 7.2 Hz, H-10b), 2.60 (1H, m, H-5), 2.54 (1H, m, H-22a), 2.46 (1H, t, *J* = 9.6 Hz, H-8), 2.33 (1H, m, H-4), 2.31 (1H, m, H-15a), 1.95 (1H, m, H-22b), 1.91 (1H, m, H-15b), 1.70 (3H, s, H_3-25), 1.50 (1H, m, H-21a), 1.43(1H, m, H-21b), 0.97 (3H, d, *J* = 6.6 Hz, H_3-24) , 0.66 (3H, d, *J* = 6.6 Hz, H_3-11); ^{13}C NMR (175 MHz, DMSO-d_6) δ_C: 208.0 (C-23), 203.8 (C-19), 173.3 (C-1), 150.0 (C-6), 147.5 (C-17), 135.9 (C-1'a), 134.7 (C-18), 132.6 (C-14), 128.8 (C-13), 127.5 (C-3'a), 124.2 (C-2'), 120.7 (C-6'), 118.7 (C-5'), 117.9 (C-4'), 111.8 (C-12), 111.3 (C-7'), 109.0 (C-3'), 70.5 (C-20), 69.5 (C-7), 61.8 (C-9), 51.6 (C-3), 48.1 (C-8), 46.2 (C-4), 40.4 (C-15), 36.1 (C-22), 32.7 (C-16), 31.9 (C-10), 31.5 (C-5), 29.8 (C-21), 20.0 (C-24), 12.9 (C-11), 11.9 (C-25).

Penochalasin G (**12**): colorless needle crystals; ^{1}H NMR (700 MHz, DMSO-d_6): δ_H 10.92 (1H, s, 1'-NH), 7.97 (1H, s, 2-NH), 7.51 (1H, d, *J* = 8.4 Hz, H-4'), 7.26 (1H, d, *J* = 8.4 Hz, H-7'), 7.09

(1H, d, J = 2.1 Hz, H-2'), 6.98 (1H, t, J = 7.7 Hz, H-6'), 6.94 (1H, t, J = 7.7 Hz, H-5'), 5.90 (1H, ddd, J = 14.7, 9.8, 2.1 Hz, H-13), 5.28 (1H, br s, H-7), 5.19 (1H, d, J = 9.8 Hz, H-17), 4.93 (1H, ddd, J = 14.7, 11.2, 2.8 Hz, H-14), 4.84 (1H, d, J = 4.2 Hz, 19-OH), 4.22 (1H, d, J = 4.2 Hz, H-19), 3.30 (1H, m, H-3), 2.82(1H, dd, J = 14.7, 5.6 Hz, H-10a), 2.63 (1H, overlapped, H-10b), 2.66 (1H, m, H-4), 2.65 (1H, m, H-8), 2.40 (1H, m, H-15a), 1.78 (1H, m, H-5), 2.14 (1H, m, H-15b), 1.95 (1H, m, H-21a), 1.90 (1H, m, H-21b), 2.35 (1H, m, H-22a), 1.23 (1H, m, H-22b), 1.70 (3H, s, H_3-25), 1.08 (3H, d, J = 7.2 Hz, H_3-11), 1.28 (3H, s, H_3-12), 0.90 (3H, d, J = 7.2 Hz, H_3-24) ; ^{13}C NMR (175 MHz, DMSO-d_6) δ_C: 209.4 (C-23), 208.9 (C-20), 173.5 (C-1), 139.2 (C-6), 137.4 (C-17), 135.9.0 (C-1'a), 131.8 (C-18), 130.5 (C-14), 130.4 (C-13), 127.8 (C-3'a), 125.4 (C-7), 125.2 (C-2'), 120.6 (C-6'), 118.6 (C-5'), 118.5 (C-4'), 111.3 (C-7'), 108.4 (C-3'), 81.4 (C-19), 66.4 (C-9), 53.2 (C-3), 49.5 (C-4), 46.6 (C-8), 41.2 (C-15), 35.0 (C-21), 34.7 (C-5), 33.9 (C-10), 31.7 (C-22), 31.6 (C-16), 21.1 (C-24), 19.8 (C-12), 13.2 (C-11), 10.7 (C-25).

Armochaetoglobin G (**13**): white amorphous powder; ^1H NMR (700 MHz, DMSO-d_6): δ_H 10.89 (1H, s, 1'-NH), 7.97 (1H, s, 2-NH), 7.48 (1H, d, J = 8.4 Hz, H-4'), 7.31 (1H, d, J = 8.4 Hz, H-7'), 7.09 (1H, d, J = 2.1 Hz, H-2'), 7.03 (1H, t, J = 7.7 Hz, H-6'), 6.96 (1H, t, J = 7.7 Hz, H-5'), 6.22 (1H, ddd, J = 15.4, 9.8, 2.1 Hz, H-13), 6.17 (1H, d, J = 9.8 Hz, H-17), 5.26 (1H, br s, H-7), 5.05 (1H, ddd, J = 15.4, 11.2, 2.8 Hz, H-14), 4.74 (1H, d, J = 6.3 Hz, 20-OH), 4.49 (1H, m, H-20), 3.26 (1H, m, H-3), 2.76 (1H, dd, J = 14.7, 5.6 Hz, H-10a), 2.69 (1H, dd, J = 14.7, 4.9 Hz, H-10b), 2.73 (1H, m, H-4), 2.70 (1H, m, H-8), 2.52 (1H, m, H-15a), 2.18 (1H, m, H-5), 1.93 (1H, m, H-15b), 2.67 (1H, m, H-21a), 1.81 (1H, m, H-21b), 1.48 (1H, m, H-22a), 1.29 (1H, m, H-22b), 1.67 (3H, s, H_3-25), 0.96 (3H, d, J = 7.2 Hz, H_3-11), 1.65 (3H, s, H_3-12), 0.90 (3H, d, J = 7.2 Hz, H_3-24) ; ^{13}C NMR (175 MHz, DMSO-d_6) δ_C: 209.3 (C-23), 203.9 (C-19), 173.7 (C-1), 147.3 (C-17), 139.1 (C-6), 136.0 (C-1'a), 134.7 (C-18), 130.7 (C-14), 130.7 (C-13), 127.7 (C-3'a), 125.8 (C-7), 124.6 (C-2'), 120.8 (C-6'), 118.4 (C-5'), 118.2 (C-4'), 111.3 (C-7'), 108.9 (C-3'), 70.9 (C-20), 66.6 (C-9), 53.4 (C-3), 49.5 (C-4), 45.6 (C-8), 40.3 (C-15), 37.7 (C-21), 32.4 (C-10), 34.4 (C-5), 32.8 (C-16), 30.1 (C-22), 19.8 (C-24), 19.7 (C-12), 13.1 (C-11), 12.1 (C-25).

Prochaetoglobosin I (**14**):^1H-NMR (500 MHz, $CDCl_3$): δ_H 5.60 (1H, s, 2-NH), 3.05 (1H, m, H-10a), 2.60 (1H, m, H-10b), 3.30 (1H, m, H-3), 3.10 (1H, t, J = 3.9 Hz, H-4), 2.50 (1H, m, H-5), 1.34 (3H, d, J = 6.6 Hz, H_3-11), 1.78 (3H, s, H_3-12), 5.37 (1H, d, J = 1.4 Hz, H-7), 2.65 (1H, dd,

J = 10.0, 1.4 Hz, H-8), 6.11 (1H, dd, *J* = 14.0, 10.0 Hz, H-13), 5.15 (1H, m, H-14), 2.25 (1H, m, H-15a), 1.94 (1H, m, H-15b), 2.45 (1H, m, H-16), 0.92 (3H, d, *J* = 6.6 Hz, H_3-24), 4.99 (1H, d, *J* = 9.0 Hz, H-17), 1.52 (1H, s, H_3-25), 2.40 (1H, m, H-19a), 2.15 (1H, m, H-19b), 2.41 (1H, m, H-20a), 2.39 (1H, m, H-20b), 6.80 (1H, m, H-21), 7.10 (1H, d, *J* = 14.0 Hz, H-22); ^{13}C NMR (125 MHz, $CDCl_3$) δ_C: 197.1 (C-23), 36.0 (C-19), 173.9 (C-1), 139.8 (C-6), 139.9 (C-17), 130.6 (C-18), 132.7 (C-14), 129.9 (C-13), 20.1 (C-12), 28.8 (C-20), 126.7 (C-7), 66.0 (C-9), 53.9 (C-3), 47.2 (C-8), 50.2 (C-4), 41.2 (C-15), 128.5(C-22), 32.4 (C-16), 35.0 (C-10), 34.8 (C-5), 146.5 (C-21), 14.3 (C-11), 21.6 (C-24), 15.6 (C-25).

Chaetoglobosin V_b (**15**): white amorphous powder; ^{1}H NMR (700 MHz, DMSO-*d*$_6$): δ_H 10.93 (1H, s, 1'-NH), 8.08 (1H, s, 2-NH), 7.38 (1H, d, *J* = 7.7 Hz, H-4'), 7.31 (1H, d, *J* =7.7 Hz, H-7'), 7.09 (1H, d, *J* = 2.1 Hz, H-2'), 7.34 (1H, t, *J* = 7.7 Hz, H-6'), 7.06 (1H, t, *J* = 7.7 Hz, H-5'), 6.96 (1H, dd, *J* = 16.1, 9.8 Hz, H-13), 5.93 (1H, ddd, *J* = 16.1, 11.0, 3.9 Hz, H-14), 5.42 (1H, d, *J* = 9.8 Hz, H-7), 4.99 (1H, d, *J* = 5.5 Hz, H-22b), 4.96 (1H, m, H-3), 3.64 (1H, m, H-4), 3.38 (1H, overlapped, H-10a), 2.70 (1H, overlapped, H-10b), 2.70 (1H, overlapped, H-8), 2.45 (1H, m, H-21), 2.27 (1H, dd, *J* =15.5, 3.2 Hz, H-22a), 2.12 (1H, m, H-16), 2.39 (1H, d, *J* = 7.3 Hz, H-17), 2.08 (1H, d, *J* = 7.3 Hz, H-17), 2.07 (1H, d, *J* =12.5 Hz, H-15a), 1.92 (1H, d, *J* = 12.5 Hz, H-15b), 1.54 (3H, s, H_3-25), 1.37 (3H, s, H_3-12), 1.50 (1H, m, H-16), 1.19 (3H, s, H_3-11), 0.96 (3H, d, *J* = 7.0 Hz, H_3-24); ^{13}C NMR (175 MHz, DMSO-*d*$_6$) δ_C: 211.4 (C-23), 203.0 (C-20), 174.1 (C-1), 149.6 (C-18), 147.2 (C-19), 136.1 (C-1'a), 133.7 (C-6), 132.4 (C-14), 130.8 (C-13), 127.1 (C-3'a), 125.6 (C-5), 123.6 (C-2'), 121.0 (C-6'), 118.5 (C-5'), 118.0 (C-4'), 111.5 (C-7'), 109.9 (C-3'), 68.4 (C-7), 65.0 (C-9), 57.3 (C-3), 53.7 (C-8), 52.9 (C-17), 49.9 (C-21), 49.5 (C-4), 44.0 (C-15), 43.4 (C-22), 42.3 (C-16), 31.9 (C-10), 21.5 (C-24), 16.9 (C-11), 16.7 (C-25), 14.5 (C-12).

Chaetoglobosin Y (**16**): white amorphous powder; $[\alpha]_D^{25}$ −21.3 (*c* 0.15, MeOH); ECD (0.2 mg/mL, MeOH) λ_{max} ($\Delta\varepsilon$) 226 (−9.8), 262 (+2.1), 304 (−3.7); ^{1}H NMR (500 MHz, $CDCl_3$) δ_H: 7.47 (1H, d, *J* = 8.0 Hz, H-4′), 7.40 (1H, d, *J* = 8.0 Hz, H-6′), 7.24 (1H, t, *J* = 7.5 Hz, H-5′), 7.16 (1H, d, *J* = 7.5 Hz, H-6′), 7.06 (1H, s, H-3′), 6.15 (1H, d, *J* = 9.0 Hz, H-17), 6.08 (1H, dd, *J* = 9.5, 13.5 Hz, H-14), 5.15 (1H, d, *J* = 11.5 Hz, H-13), 4.76 (1H, s, H-20), 3.75 (1H, m, H-3), 3.66 (1H, d, *J* = 9.5 Hz, H-8), 3.07 (1H, d, *J* = 12.5 Hz, H-10a), 2.72 (2H, m, H-10b, H-16), 2.66 (2H, m, H-22), 2.61 (1H, m, H-4), 2.44 (1H, d, *J* = 13.0 Hz), 2.26 (1H, m, H-5), 2.12 (3H, m, H-6,

H-15), 1.92 (1H, m, H-21a), 1.84 (3H, s, H-25), 1.70 (1H, m, H-21b), 1.17 (3H, d, *J* = 3.0 Hz, H-11), 1.15 (3H, d, *J* = 3.0 Hz, H-12), 1.03 (3H, d, *J* = 6.5 Hz, H-24); ^{13}C NMR (125 MHz, $CDCl_3$) δ_C: 212.4 (C-7), 206.6 (C-23), 203.5 (C-19), 172.8 (C-1), 149.1 (C-17), 136.4 (C-1′a), 134.4 (C-14), 134.1 (C-18), 127.0 (C-3′a), 124.8 (C-13), 123.3 (C-2′), 122.6 (C-6′), 120.1 (C-5′), 118.2 (C-4′), 111.6 (C-7′), 110.4 (C-3′), 71.6 (C-20), 64.4 (C-9), 52.7 (C-3), 52.1 (C-8), 47.4 (C-4), 46.4 (C-6), 40.8 (C-15), 36.7 (C-22), 35.1 (C-5), 33.3 (C-16), 31.6 (C-21), 34.3 (C-10), 19.8 (C-24), 16.1 (C-12), 15.7 (C-11), 12.3 (C-25); HR-ESIMS *m*/*z* 531.2849 (calcd for $C_{32}H_{39}N_2O_5$, 531.2853), 553.2664 (calcd for $C_{32}H_{38}N_2O_5Na$, 553.2673).

Table S3. Cytotoxicity activities of compounds **3**–**16**. (IC_{50}, μM)

NO.	K562	A549	Huh7	H1975	MCF-7	U937	BGC823	HL60	Hela	MOLT-4
**3**	> 10	> 10	> 10	> 10	> 10	> 10	> 10	> 10	> 10	> 10
**4**	> 10	> 10	> 10	> 10	> 10	> 10	> 10	> 10	> 10	> 10
**5**	> 10	> 10	> 10	> 10	> 10	> 10	> 10	> 10	> 10	> 10
**6**	8.9	5.9	1.4	9.2	2.1	1.4	8.2	2.5	2.8	1.4
**7**	> 10	> 10	> 10	> 10	> 10	> 10	> 10	> 10	> 10	> 10
**8**	> 10	> 10	> 10	> 10	> 10	> 10	> 10	> 10	> 10	> 10
**9**	> 10	> 10	> 10	> 10	> 10	> 10	> 10	> 10	> 10	> 10
**10**	> 10	> 10	> 10	> 10	> 10	> 10	> 10	> 10	> 10	> 10
**11**	> 10	> 10	3.0	> 10	7.5	4.9	> 10	> 10	> 10	2.9
**12**	> 10	> 10	> 10	> 10	> 10	> 10	> 10	> 10	> 10	> 10
**13**	> 10	> 10	> 10	> 10	> 10	> 10	> 10	> 10	> 10	> 10
**14**	> 10	> 10	> 10	> 10	> 10	> 10	> 10	> 10	> 10	> 10
**15**	> 10	> 10	> 10	> 10	> 10	> 10	> 10	> 10	> 10	> 10
**16**	> 10	> 10	> 10	> 10	> 10	> 10	> 10	> 10	> 10	> 10

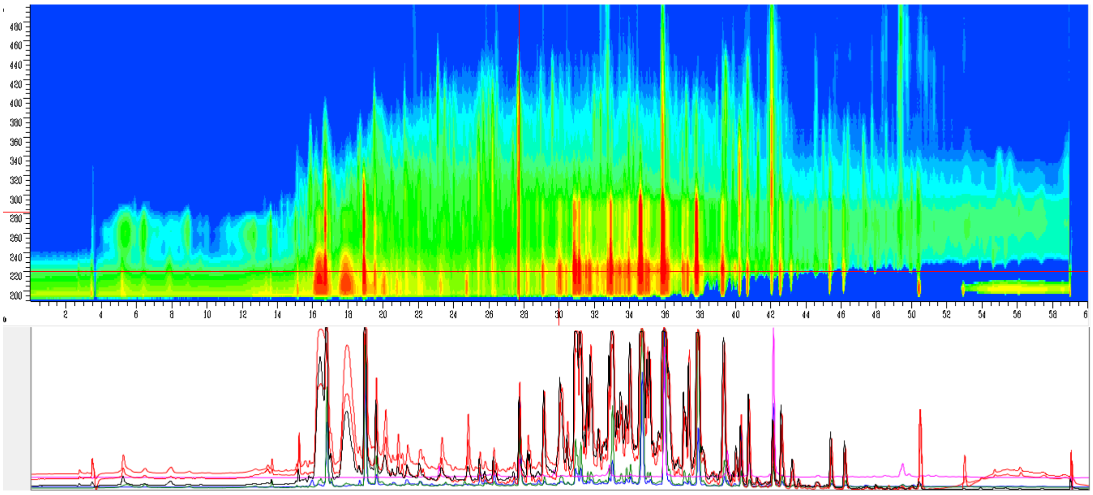

Figure S12. HPLC profiles of the EtOAc extracts of *Chaetomium globosum* C2F17.

Figure S13. The ITS sequence of *Chaetomium globosum* C2F17

tttccgtaggggggacctGCGGAGGGATCATTACAGAGTTGCAAAACTCCCTAAACCATTGT

GAACGTTACCTATACCGTTGCTTCGGCGGGCGGCCCCGGGGTTTACCCCCCGGGC

GCCCCTGGGCCCCACCGCGGGCGCCCGCCGGAGGTCACCAAACTCTTGATAATTT

ATGGCCTCTCTGAGTCTTCTGTACTGAATAAGTCAAAACTTTCAACAACGGATCTC

TTGGTTCTGGCATCGATGAAGAACGCAGCGAAATGCGATAAGTAATGTGAATTGC

AGAATTCAGTGAATCATCGAATCTTTGAACGCACATTGCGCCCGCCAGCATTCTG

GCGGGCATGCCTGTTCGAGCGTCATTTCAACCATCAAGCCCCCGGGCTTGTGTTG

GGGACCTGCGGCTGCCGCAGGCCCTGAAAAGCAGTGGCGGGCTCGCTGTCGCAC

CGAGCGTAGTAGCATACATCTCGCTCTGGTCGCGCCGCGGGTTCCGGCCGTTAAA

CCACCTTTTAACCCAAGGTTGACCTCGGATCAGGTAGGAAGACCCGCTGAACTTA

AGCATatataagcgcgaaggaa
